# Isocitrate dehydrogenase 1–snail axis dysfunction significantly correlates with breast cancer prognosis and regulates cell invasion ability

**DOI:** 10.1186/s13058-018-0953-7

**Published:** 2018-04-16

**Authors:** Wen-Shan Liu, Shih-Hsuan Chan, Hong-Tai Chang, Guan-Cheng Li, Ya-Ting Tu, Hui-Hwa Tseng, Ting-Ying Fu, Hui-Yu Chang, Huei-Han Liou, Luo-Ping Ger, Kuo-Wang Tsai

**Affiliations:** 1 Department of Radiation Oncology, Kaohsiung Veterans General Hospital, Kaohsiung, Taiwan; 2 School of Medicine, National Defense Medical Center, Taipei, Taiwan; 3 Institute of Molecular and Genomic Medicine, National Health Research Institutes, Zhunan, Taiwan; 4 Institute of Molecular Medicine, College of Life Science, National Tsing Hua University, Hsinchu, Taiwan; 5 Graduate Institute of Integrated Medicine, China Medical University, Taichung, Taiwan; 6 Department of Surgery, Kaohsiung Veterans General Hospital, Kaohsiung, Taiwan; 7 Department of Medical Education and Research, Kaohsiung Veterans General Hospital, Kaohsiung, 813 Taiwan, Republic of China; 8 Department of Pathology and Laboratory Medicine, Kaohsiung Veterans General Hospital, Kaohsiung, Taiwan; 9 Department of Chemical Biology, National Pingtung University of Education, Pingtung, Taiwan; 10 Institute of Biomedical Sciences, National Sun Yat-Sen University, Kaohsiung, Taiwan

## Abstract

**Background:**

The isocitrate dehydrogenase (IDH) gene family expresses key functional metabolic enzymes in the Krebs cycle and mediates the epigenetic reprogramming, which serves as an important biomarker of breast cancer. However, the expression levels of the IDH protein and their biological function in human breast cancer remain largely unknown.

**Methods:**

In this study, the clinical impact of IDH1 expression on the progression and prognosis of breast cancer was evaluated using immunohistochemistry assay (IHC) of the corresponding tumor-adjacent normal, ductal carcinoma in situ (DCIS), and invasive ductal carcinoma (IDC) tissues from 309 patients with breast ductal carcinoma. The relationship between microRNA (miRNA) and IDH1 were examined by a bioinformatics approach, western blot and reporter assay. The biological functions of IDH1 were examined in breast cancer cells with IDH1 knockdown, including proliferation, migration and invasion.

**Results:**

The present findings revealed that the mRNA and protein expression levels of IDH1 were both significantly lower in breast cancer tissues than in adjacent normal tissues. A low expression level of IDH1 in breast cancer significantly correlated with advanced stage (*p* = 0.012), lymph node metastasis (*p* = 0.018), and poor disease-specific survival (DSS) (adjusted hazard ratio (AHR), 1.57, 95% confidence interval (CI), 1.08–2.30; *p* = 0.02). Furthermore, oncogenic miR-32 and miR-92b were identified to suppress IDH1 expression, leading to the inhibition of cell migration and invasion. We further explored whether reduced expression of IDH1 significantly increases snail expression by activating HIFα (hypoxia-inducible factor-1 alpha) and NFκB (nuclear factor kappa B) signaling. Multivariate Cox regression analysis revealed that the combination of low IDH1 and high snail expression could be an independent risk factor for shorter DSS (AHR, 2.34; 95% CI, 1.32–4.16; *p* = 0.004) and shorter disease-free survival (AHR, 2.50; 95% CI, 1.39–4.50; *p* = 0.002) in patients with breast cancer.

**Conclusion:**

Our findings revealed that a IDH1^low^/Snail^high^ molecular signature could serve as an independent biomarker for poor prognosis in breast cancer

**Electronic supplementary material:**

The online version of this article (10.1186/s13058-018-0953-7) contains supplementary material, which is available to authorized users.

## Background

Breast cancer is a common cancer worldwide, and its incidence is gradually increasing in Asia. It is a heterogeneous cancer type and can be distinguished into luminal A and B, human epidermal growth factor receptor 2 overexpression, basal-like, unclassified, and various other subtypes [1–3]. These subtypes are associated with specific morphological characteristics, metastatic ability, and chemosensitivity, and yield different clinical outcomes [2–4]. However, metastasis is a major problem resulting in therapy failure and lethality in patients with breast cancer. Therefore, investigating the detailed mechanisms of breast cancer metastasis or developing a favorable prognostic biomarker to predict prognostic outcomes in patients with breast cancer is beneficial.

The isocitrate dehydrogenase (IDH) gene family expresses key functional metabolic enzymes in the Krebs cycle, which can catalyze the conversion of isocitrate to α-ketoglutarate (KG), thus generating nicotinamide adenine dinucleotide phosphate-oxidase (NADPH) from NADP+. In addition, α-KG, a cofactor for the ten–eleven translocation (TET) family, catalyzes the conversion of cytosine-5 methylation to cytosine-5 hydroxymethylation [5]. Therefore, the physiological function of the NADP-dependent IDH gene has been reported to involve the maintenance of the normal cellular redox status and global DNA methylation status in normal cells [6, 7]. Studies have identified that somatic heterozygous mutations in IDH1/2 play a crucial role in the development of cancers, including glioma and leukemia [5, 7, 8]. Zhao et al. reported that IDH1 mutations contribute to tumorigenesis by modulating the stabilization of hypoxia-inducible factor (HIF)-1 [9]. In addition, IDH1/2 mutations have frequently resulted in the accumulation of D-2-hydroxygluarate, which blocks TET-induced cytosine 5-hydroxymethylation, resulting in increased global DNA hypermethylation. In acute myeloid leukemia, IDH1/2 mutations are closely associated with poor prognosis [5, 7, 10]. On the other hand, the low frequency of IDH1/2 mutations has been reported in breast cancer [8, 11, 12]. Furthermore, the biological function and clinical effects of the IDH gene in breast cancer have not been characterized in depth. In this study, we first reported that the IDH1 is downregulated in breast cancer and depletion of IDH1 in breast cancer cells results in accelerating breast cancer migration and invasion activities by activating snail expression. Two novel micro RNAs (miRNAs), miR-32-5p and miR-92b-3p, were identified to directly inhibit IDH1 expression. Clinically, the IDH1–snail axis dysfunction might be a favorable independent marker for predicting breast cancer survival.

## Methods

### Patients and tissues

This study was approved by the institutional review board (IRB) of Kaohsiung Veterans General Hospital, Kaohsiung, Taiwan (IRB number: VGHKS13-CT10-10). The requirement for written informed consent from patients was waived by the hospital IRB because all the data and specimens were previously collected and anonymously analyzed. Tissue microarrays containing 309 paraffin-embedded samples of breast tissue from invasive ductal carcinoma (IDC) were established, and the pathological information on the patients was summarized in our previous study [13].

### Expression data from The Cancer Genome Atlas

All level-3 expression data for breast cancer were downloaded from The Cancer Genome Atlas (TCGA) database. The transcriptome data on 1102 and 113 breast cancer and adjacent normal tissues, respectively, were downloaded from the TCGA database. In addition, the small RNA profiles of 778 and 87 breast cancer and adjacent normal tissues, respectively, were obtained from TCGA database. The expression levels of protein-coding genes were shown as reads per kilo base million and those of miRNA were shown as transcripts per million.

### Immunohistochemistry analysis

A Novolink max polymer detection system (Leica, Newcastle upon Tyne, UK) was used for immunohistochemical (IHC) analysis in this study. The slides were deparaffinized in xylene and rehydrated in graded alcohol. Antigen retrieval was performed by immersing the slides in Tris-ethylenediaminetetraacetic acid (10 mM and pH 9.0) at 125 °C for 10 min in a pressure boiler. Endogenous peroxidase activity was blocked by incubating the slides with 3% hydrogen peroxide in methanol for 30 min. After blocking at room temperature, primary antibodies were immediately applied, and the slides were incubated overnight at 4 °C in a wet chamber. The following primary antibodies were used in this study: rabbit polyclonal anti-IDH1 (1:400; GeneTex, San Antonio, Texas, USA) in Tris-buffered saline solution with 5% bovine serum albumin. After being washed with phosphate-buffered saline, the slides were incubated with horseradish peroxidase-labeled secondary antibody for 10 min at room temperature, and the sections were counterstained with hematoxylin.

### IHC analysis and scoring

First, a senior pathologist accompanied a technician to evaluate the slides until all the discrepancies were resolved. Subsequently, the technician independently reviewed all the slides. Finally, 5–20% of core samples at each intensity were randomly selected for re-evaluation by the pathologist. During the evaluation, the pathologist and technician were blinded to the clinical outcomes of the patients. We graded the immunoreactivity using a semiquantitative approach. Marker scores for staining were calculated based on the staining intensity (0, no signal; 1, mild; 2, moderate; and 3, strong) and the proportion of positively stained tumor cells in the 5 high-power field (0, < 5%; 1, 5–25%; 2, 26–50%; 3, 51–75%; and 4, > 75%). The marker score is the sum of the staining intensity score and percentage of the positive tumor cell score. The score was graded as follows: –, 0–1; +, 2–3; ++, 4–5; and +++, 6–7.

### Real-time reverse transcription polymerase chain reaction

Two micrograms of total RNA was reverse-transcribed with oligo (dT)_15_ primers and SuperScript III Reverse Transcriptase according to the manufacturer’s instructions (Invitrogen, Carlsbad, CA, USA). The reaction was performed by incubating cells at 42 °C for 1 h; the Reverse Transcriptase was subsequently inactivated by incubation at 85 °C for 5 min. The cDNA was used for real-time polymerase chain reaction (PCR) analysis with gene-specific primers, and gene expression was detected using a SYBR Green I assay (Applied Biosystems, Foster City, CA, USA). To determine their expression levels, the genes were subjected to the following conditions: 94 °C for 10 min, followed by 40 cycles of 94 °C/1 min, 60 °C/1 min, and 72 °C/30 s, with a final extension at 72 °C for 10 min. GAPDH expression was used as an internal control, and the expression levels of epithelial–mesenchymal transition (EMT)-relative genes were normalized to those of glyceraldehyde-3-phosphate dehydrogenase (GAPDH) (difference in cycle threshold (ΔCt) = Ct_candidates_ − Ct_GAPDH_). The real-time PCR primers used in this study are listed in Additional file 1: Table S1.

### Stem-loop reverse transcription PCR

We reverse-transcribed 1 μg of total RNA with a stem-loop reverse transcription (RT) reaction using microRNA RT primers and SuperScript III Reverse Transcriptase according to the manufacturer’s instructions (Invitrogen, Carlsbad, CA, USA). The reaction was performed under the following incubation conditions: 30 min at 16 °C, followed by 50 cycles of 20 °C/30 s, 42 °C/30 s, and 50 °C/1 s. The enzyme was subsequently inactivated by incubation at 85 °C for 5 min. Gene expression was detected using the SYBR Green I assay (Applied Biosystems, Foster City, CA, USA), and the expression levels of microRNA were normalized to those of U6 small RNA (ΔCt = target miRNA Ct-U6 Ct).

### Ectopic expression of miRNAs

Breast cancer cells were transfected with 10-nM miRNA-32-5p mimics, miR-92b-3p mimics, or the appropriate miRNA mimic control (GenDiscovery Biotechnology Inc., Taiwan) using the Lipofectamine RNAiMAX reagent (Invitrogen, Carlsbad, CA, USA). Twenty-four hours after transfection, cells were harvested, and their expression levels were examined using stem-loop RT quantitative PCR.

### Knockdown of IDH1 expression

Breast cancer cells were transfected with 10-nM si-IDH1#1 (sense: 5′- CAUUAAAGGUUUACCCAAUtt-3′ and antisense: 5′- AUUGGGUAAACCUUUAAUGca-3′, si-IDH1#2 (sense: 5′- CCAACGACCAAGUCACCAAtt-3′ and antisense: 5′- UUGGUGACUUGGUCGUUGGtg-3′) or scramble control (Invitrogen, Carlsbad, CA, USA) using the Lipofectamine RNAiMAX reagent (Invitrogen, Carlsbad, CA, USA). Twenty-four hours after transfection, cells were harvested, and their expression levels were examined through western blotting.

### Stable IDH1 knockdown with short hairpin RNA (shRNA)

Stable MDA-MB-231 cells with IDH1 knockdown were generated by infecting MDA-MB-231 cells with lentiviruses expressing sh-IDH1 in the presence of 8 μg/mL of polybrene for 24 h, followed by puromycin (4 μg/mL) selection for 3–5 days. The shLuc vector targeting the luciferase gene provided puromycin resistance and was used as the control. IDH1 expression was confirmed through a western blotting assay. In this study, we designed two shRNA sequences targeting IDH1, and the individual sequences of shRNA used for constructs in this study were as follows: for sh-IDH1 #1, sense: 5′-CCGGCCTATCATCATAGGTCGTCATCTCGAGATGACGACCTATGATGATAGGTTTTT-3′ and antisense: 5′-AAAAACCTATCATCATAGGTCGTCATGAGCTCATGACGACCTATGATGATAGGCCGG-3′; for sh-IDH2#2, sense: 5′-CCGGGCTTTGGAAGAAGTCTCTATTCTCGAGAATAGAGACTTCTTCCAAAGCTTTTT-3′ and antisense: 5′-AAAAAGCTTTGGAAGAAGTCTCTATTGAGCTCAATAGAGACTTCTTCCAAAGCCCGG-3′.

### Cell proliferation assays

Breast cancer cells (2.5 × 10^3^ cells/mL) were seeded in a 96-well plate and transfected with siIDH1, shIDH1 or scramble control. After transfection, cell growth was determined at 0, 1, 2, 3, and 4 days using the CellTiter-Glo® One Solution Assay (Promega Corporation, Madison, WI, USA). All the experiments were performed in triplicate.

### Wound healing assay

For the wound healing assay, after transfection with si-IDH1 or scramble control for 24 h, cells (1.5 × 10^6^) were seeded on six-well plates. A straight line was scratched on the monolayer in the middle of the well using the tip of a 200-mL pipette. A culture medium of 10% fetal bovine serum (FBS) was replaced with a serum deprivation culture medium, and cells were then incubated at 37 °C. Wound closure was monitored and photographed at different time points under a microscope. Subsequently, the open area was assessed for quantifying cell migration ability.

### Invasion assays

Cells were assessed for their invasion ability in vitro using a Transwell assay, according to our previous study [14]. Briefly, breast cancer cells (4.5 × 10^5^) transfected with si-IDH1, miR-32-5p mimics, miR-92b-3p mimics, or scramble control were suspended in 2% FBS and seeded on the upper chamber of the Transwells (Falcon, Corning Incorporated, USA) with a coating of Matrigel (BD Biosciences, MA, USA) for the invasion assay. After incubating in a CO_2_ incubator at 37 °C for 12 or 24 h, the remaining cells in the upper chamber were removed with cotton swabs, and cells on the undersurface of the Transwells were fixed with 10% formaldehyde solution. Cells were stained with crystal violet solution, and the numbers of breast cancer cells were calculated by counting three fields under a phase-contrast microscope. All experiments were performed in triplicate.

### Candidates of the miRNA target and assay of luciferase activity

The putative miRNAs targeting the 3′-UTR of IDH1 were determined using TargetScan (http://www.targetscan.org/vert_71/) and microRNA.org (http://34.236.212.39/microrna/home.do). In this study, we identified that 15 miRNAs were predicted for binding at the 3′-UTR of IDH1. The full-length 3′-UTR of IDH1 was cloned into the PGL3 vector. Subsequently, pGL3–IDH1–3′-UTR vectors were co-transfected with miR-32-5p mimics, miR-92b-3p mimics, or scramble controls in breast cancer cells using the Lipofectamine RNAiMAX reagent (Invitrogen, Carlsbad, CA, USA). After transfection for 24 h, cell lysates were used for measuring the luciferase activity by using the Dual-Glo Luciferase Reporter Assay System (Promega Corporation, Madison, WI, USA).

### Western blotting

Total cell lysates were extracted with radioimmunoprecipation assay (RIPA) buffer (50 mM Tris-HCl at pH 8.0, 150 mM NaCl, 1% NP-40, 0.5% deoxycholic acid, and 0.1% sodium dodecyl sulfate). Total proteins were separated through 6–10% sodium dodecyl sulfate–polyacrylamide gel electrophoresis and transferred onto nitrocellulose filter membranes (Millipore, Billerica, USA). After the transfer, the membranes were blocked with a blocking buffer for 1 h at room temperature and probed with a primary antibody for the desired molecule overnight at 4 °C, followed by treatment with horseradish peroxidase-conjugated secondary antibody for 1 h at room temperature. Finally, the proteins were visualized using WesternBright™ ECL (Advansta Inc., Menlo Park, CA, USA) and detected using the BioSpectrum^TW^ 500 Imaging System (UVP, USA). The antibodies are shown in Additional file 2: Table S2.

### Microarray analysis and pathway enrichment analysis

Total RNA samples were obtained from si-IDH1 and scramble control cells and were subjected to microarray screening. The microarray experiments and data analysis were conducted by Welgene Biotech (Taipei, Taiwan) through the Agilent SurePrint G3 Human V2 GE 8X60K Chip. The differentially expressed genes (fold change > 2) were selected from microarray data, and the candidate genes were mapped onto the Kyoto Encyclopedia of Genes and Genomes pathways based on the enzyme commission numbers by using the R package SubPathwayMiner v.3.1. Subsequently, the hypergeometric test was performed to identify significantly enriched pathways and calculate the false-positive discovery rate in terms of the *p* value.

### Metabolite analysis

α-KG was analyzed through ultra-high performance liquid chromatography (UPLC; Acquity UPLC system, Waters Corporation, MA, US) coupled with a quadrupole time-of-flight mass spectrometer (QTof MS) (Xevo, Waters). Details of the procedures were described in another study [15]. Raw data on all samples were processed using MassLynx software.

### Protein degradation assay

Cells subjected to different treatments were cultured in the presence of 20 μg/mL of cyclohexamide for the indicated time periods and then harvested in RIPA buffer containing protease inhibitors (Roche, CA, USA). Protein lysates were quantified using the bicinchoninic acid (BCA) method for the subsequent western blotting analysis.

### Statistical analysis

Many statistical methods were used for data analysis. The chi-squared test, Student *t* test, analysis of variance (ANOVA), Mann–Whitney U test, and Kruskal–Wallis one-way ANOVA were used to test correlation between the expression levels of each protein and different types of breast tissues or clinicopathological parameters. Studies on breast cancer have typically defined the outcomes as the time from diagnosis or surgery until a particular event of interest (endpoint). Disease-specific survival (DSS) is measured from the time of the initial resection of the primary tumor to the date of cancer-related death or last follow up. Disease-free survival (DFS) is defined as the time from surgery to an event (local recurrence, regional recurrence, and distant metastasis, but not disease-related death). The cumulative survival curves were estimated using the Kaplan–Meier method, and the survival curves were compared using the log-rank test. A Cox proportional hazards model was used to determine the independent predictors of survival using factors significant in univariate analysis as covariates. We considered *p* < 0.05 (two-sided) as significant.

## Results

### IDH1 was significantly downregulated in breast cancer

**Fig. 1:**
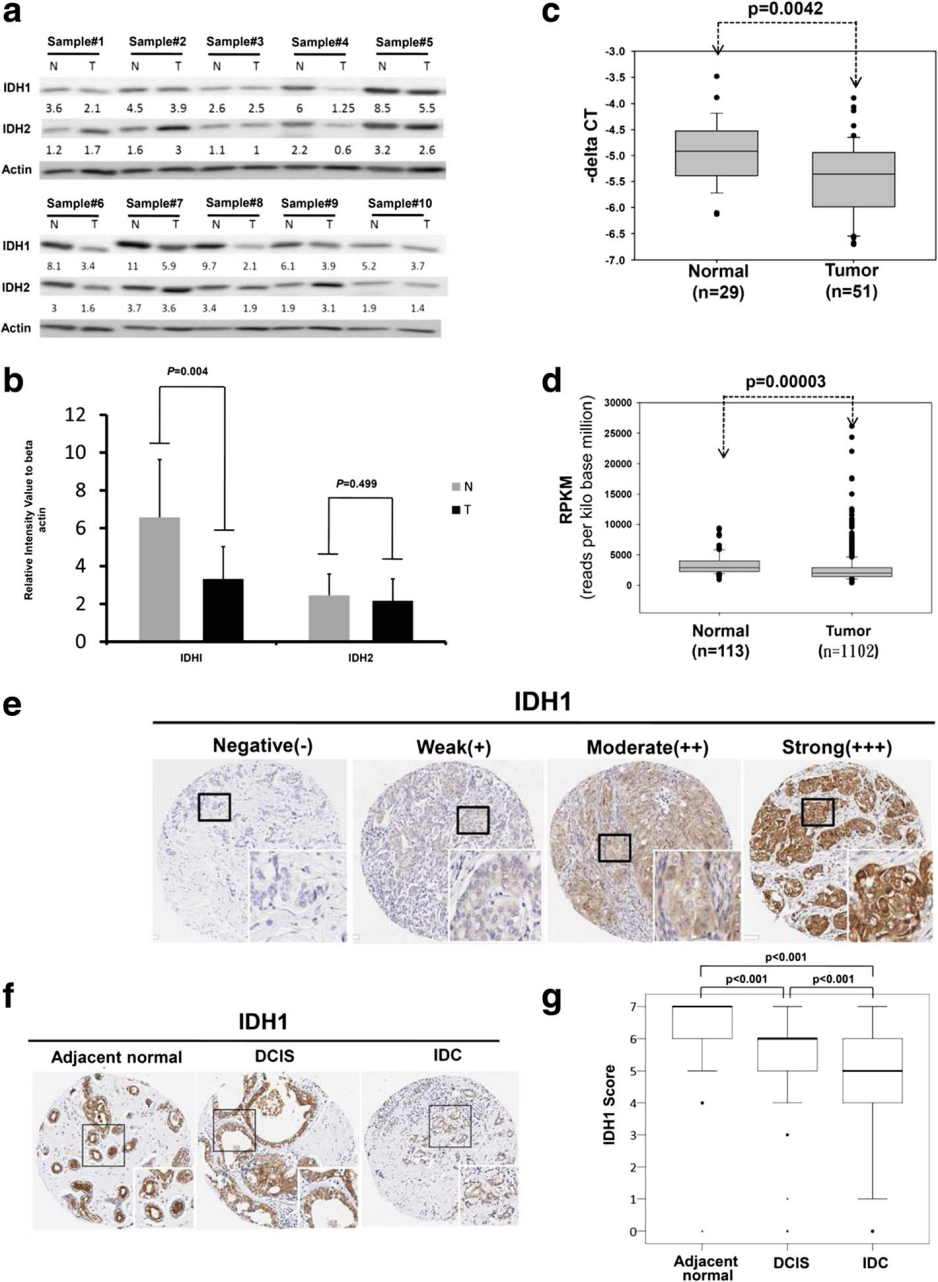
Isocitrate dehydrogenase 1 (IDH1) expression was significantly lower in breast cancer **a** Protein levels of IDH1 and IDH2 in breast cancer were examined and matched through western blotting with those in adjacent normal tissues obtained from 10 patients. **b** Expression levels of IDH1 and IDH2 were further quantified using Image J. N, normal; T, tumor. **c** Expression levels of IDH1 were assessed in breast cancer tissues (n = 51) and adjacent normal tissues (n = 29) using a real-time PCR approach. CT, cycle threshold. **d** Expression levels of IDH1 transcripts were analyzed in breast cancer tissues (n = 1102) and compared with those in adjacent normal tissues (n = 113) from The Cancer Genome Atlas database. **e** Immunohistochemistry (IHC) analysis of IDH1 expression in the breast cancer tissue microarray from 309 patients. Representative photomicrographs are presented. Representative photomicrographs showed negative (−), weak (+), moderate (++), and strong (+++) staining in invasive ductal carcinoma (IDC) tissues. **f**, **g** IDH1 levels were significantly lower in ductal carcinoma in situ and IDC tissues compared with adjacent normal tissues

First, we examined the expression levels of IDH1 and IDH2 in breast cancer tissues and adjacent normal tissues from 10 patients using western blotting. The results revealed that IDH1 expression levels were significantly lower in breast cancer tissues than in adjacent normal tissues (*p* = 0.004; Fig. 1a and b). However, IDH2 expression levels did not differ significantly. We further assessed the mRNA expression levels of IDH1 using a real-time PCR approach, which revealed that IDH1 expression levels and transcript levels were significantly lower in breast cancer tissues than in adjacent normal tissues (*p* = 0.0042; Fig. 1c). By analyzing TCGA database, we observed the same result that IDH1 expression levels were significantly lower in breast cancer compared to adjacent normal tissues (*p* = 0.00003; Fig. 1d). To assess whether the lower IDH1 expression levels contributed to breast cancer progression, we performed IHC analysis of tissue microarrays containing adjacent normal tissues (n = 309), ductal carcinoma in situ (DCIS) tissues (*n* = 120), and IDC tissues (n = 309) obtained from 309 patients with breast cancer. The results revealed that IDH1was expressed in the cytoplasm; representative photomicrographs show negative (−), weak (+), moderate (++), and strong (+++) staining in IDC tissues (Fig. 1e). The IHC data revealed that IDH1 expression levels were significantly lower in DCIS tissues (*p* < 0.001) and IDC tissues (*p* < 0.001) than in adjacent normal tissues. Furthermore, IDH1 expression levels were significantly lower in IDC tissues than in DCIS tissues (*p* < 0.001; Fig. 1f and g). Together, these results indicated that the protein expression levels of IDH1 were gradually decreased during breast cancer progression.

### Low IDH1 expression levels were associated with poor clinicopathological features

**Fig. 2:**
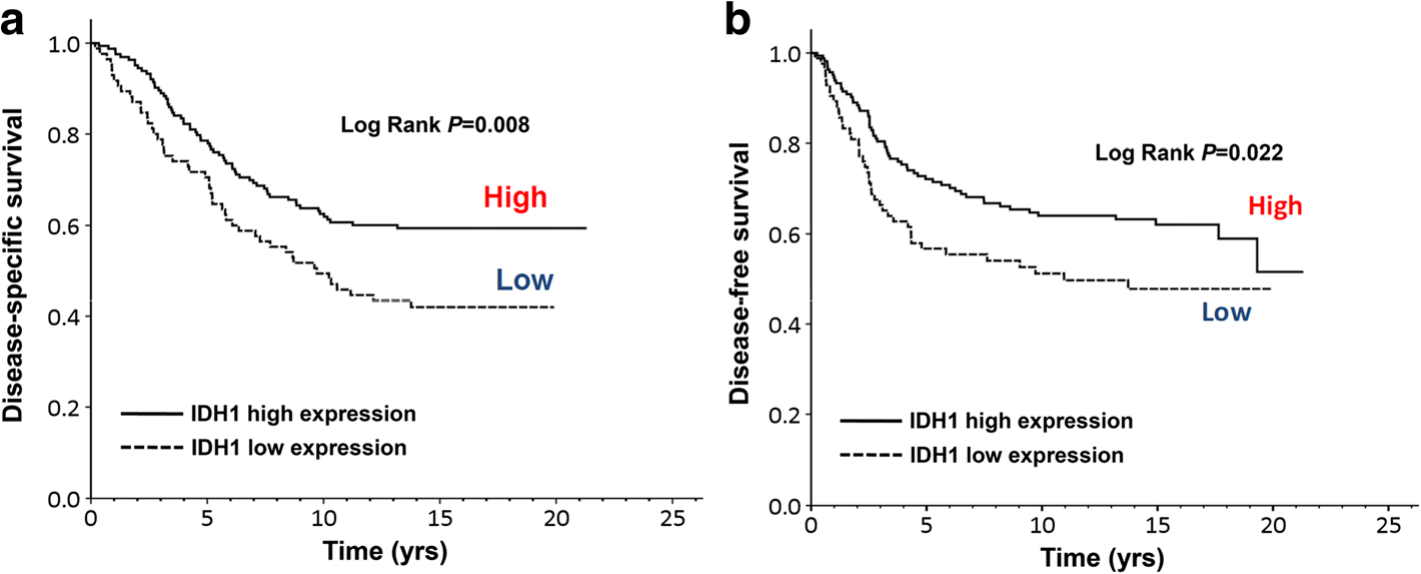
Isocitrate dehydrogenase 1 (IDH1) expression levels closely correlated with the survival curve of patients with breast cancer. **a**, **b** Disease-specific survival and disease-free survival were compared according to IDH1 expression in breast cancer using the log-rank test

We investigated whether IDH1 protein dysfunction contributes to the clinicopathological features of breast cancer. As shown in Table 1, low IDH1 expression levels were significantly correlated with the early pathology stage (*p* = 0.012) and early perineural invasion (pN) stage (*p* = 0.018). Furthermore, high IDH1 expression levels were significantly correlated with human epidermal growth factor receptor (HER)-positive human breast cancer (*p* = 0.046), whereas no difference was observed in estrogen receptor (ER) and progesterone receptor (PR) status (Additional file 3: Table S3). Univariate logistic analysis revealed that low IDH1 expression levels resulted in poor DSS (crude hazard ratio (CHR), 1.64; 95% confidence interval (CI), 1.14–2.38; *p* = 0.008) and DFS (CHR, 1.58; 95% CI 1.07–2.34; *p* = 0.023) in patients with breast cancer (Table 2 and Fig. 2). Multivariate logistic analysis and plotting of survival curves showed that low IDH1 expression levels were significantly associated with poor DSS (adjusted HR (AHR), 1.57; 95% CI, 1.08–2.30; *p* = 0.02) but not DFS (AHR, 1.40; 95% CI, 0.93–2.10; *p* = 0.106) in patients with breast cancer (Table 2). An analysis of TCGA database revealed that low IDH1 mRNA expression levels were significantly associated with poor overall survival in patients with breast cancer (CHR, 1.80; 95% CI% 1.10-2.94; *p* = 0.02) (Additional file 4: Table S4). These data suggest that IDH1 might be involved in the growth, migration, and invasion of breast cancer cells and that low IDH1 expression correlates with poor prognosis in patients with breast cancer. In addition, several studies have showed that the IHC antibody raised against mutant IDH1 protein with R132H mutation can specifically recognize this mutant protein in the tumor tissues using immunochemistry staining (IHC). Therefore, we examined the presence of R132H mutant IDH1 in breast cancer tissues using the IHC approach, and this mutation was absent in breast cancer tissue from the 309 women (data not shown). This finding was in accordance with a previous study [12]. Together, our data revealed that aberrantly low expression levels of IDH1, rather than IDH1 mutation, had crucial effects on the prognosis of breast cancer.

**Table 1:** Correlation of ΙDΗ1 expression with clinicopathological characteristics of patients with IDC of the breast

Variables	IDH1			
	%	Mean ± SD	Median	*p* value
Age (years)				
<40	16.9	4.07 ± 2.13	5.00	0.068^a^
40–59	55.1	4.85 ± 1.87	6.00	
≧60	28.1	4.67 ± 2.06	5.00	
Menopausal status				
Perimenopausal and premenopausal	47.6	4.51 ± 2.00	5.00	0.225^b^
Postmenopausal	52.4	4.80 ± 1.97	6.00	
Grading				
Well-differentiated + moderately differentiated	77.2	4.79 ± 1.95	5.00	0.070^b^
Poor	22.8	4.26 ± 2.06	4.00	
Pathology stage				
I	15.7	5.38 ± 1.55^d, e^	6.00	***0.012*** ^c^
II	49.1	4.64 ± 2.02^d^	5.00	
III	35.2	4.38 ± 2.05^e^	5.00	
pT stage				
T1	25.1	4.93 ± 1.79	5.00	0.186^a^
T2	64.4	4.66 ± 2.01	5.00	
T3 + T4	10.5	4.11 ± 2.20	4.50	
pN stage				
N0	40.4	5.06 ± 1.78^f, g^	6.00	***0.018*** ^c^
N1	26.6	4.34 ± 2.06^f^	5.00	
N2	22.8	4.26 ± 2.24^g^	5.00	
N3	10.1	4.85 ± 1.73	5.00	
Vascular invasion				
Absent	62.9	4.73 ± 1.95	5.50	0.524^b^
Present	37.1	4.57 ± 2.05	5.00	
Nipple invasion				
Absent	90.6	4.69 ± 2.00	5.00	0.622^b^
Present	9.4	4.48 ± 1.83	5.00	

*IDH* isocitrate dehydrogenase, *IDC* invasive ductal carcinoma

^a^*p* values were estimated by one-way analysis of variance (ANOVA)

^b^*p* values were estimated by Student’s *t* test

^c^*p* values were estimated by Kruskal–Wallis one-way ANOVA

^d^*p* = 0.027, stage I compared with stage II

^e^*p* = 0.003, stage I compared with stage III

^f^*p* = 0.006, stage N0 comapred with stage N1

^g^*p* = 0.013, stage N0 comapred with stage N2

Bold italics values denote statistically significant

**Table 2:** Univariate and multivariate Cox regression analysis of IDH1 expression for disease-specific survival and disease-free survival of patients with breast IDC

Characteristic	Number (percentage)	DSS				DFS			
		CHR^a^ (95% CI)	*p* value	AHR^b^ (95% CI)	*p* value	CHR^a^ (95% CI)	*p* value	AHR^b^ (95% CI)	*p* value
IDH1 status	(n = 249)								
High (5–7)	164 (65.9)	1.00		1.00		1.00		1.00	
Low (0–4)	85 (34.1)	1.64 (1.14–2.38)	***0.008***	1.57 (1.08–2.30)	***0.020***	1.58 (1.07–2.34)	***0.023***	1.40 (0.93–2.10)	0.106

*Abbreviations*: *IDH* isocitrate dehydrogenase, *IDC* invasive ductal carcinoma, *DSS* disease***-***specific survival, *DFS* disease***-***free survival, *CHR* crude hazard ratio, *AHR* adjusted hazard ratio

^a^CHRs were estimated by univariate Cox regression

^b^AHRs were adjusted for American Joint Committee on Cancer pathological stage (II and III vs. I), grading (III vs. I and II), incomplete or inappropriate adjuvant treatment vs. non-treatment or complete adjuvant treatment, and molecular subtypes (luminal B, human epidermal growth factor receptor 2 overexpression and basal-like vs. luminal A) in multivariate Cox regression

Bold italic values denote statistically significant

### Oncogenic miR-32-5p and miR-92b-3p suppressed IDH1 expression to enhance invasion ability in breast cancer

**Fig. 3:**
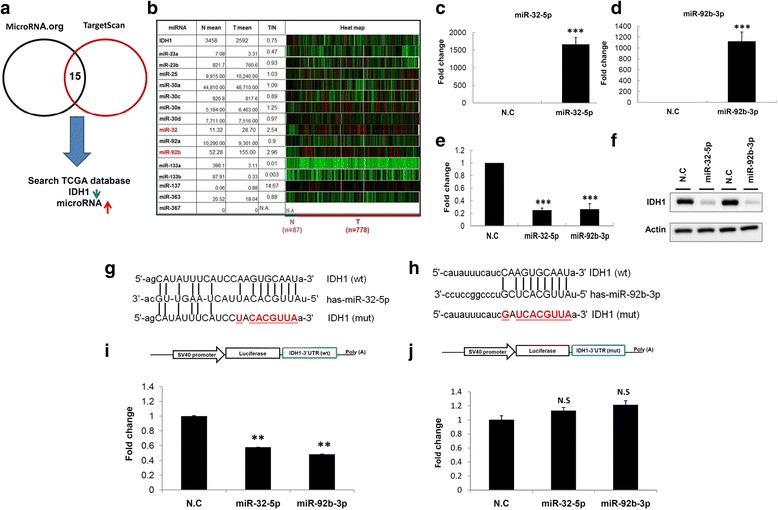
miR-32-5p and miR-92b-3p suppressed isocitrate dehydrogenase (IDH1) expression by directly targeting its 3′-UTR in breast cancer cells. **a** The 15 putative miRNA candidates that were predicted for targeting the 3′-UTR of IDH1 using TargetScan and MicroRNA.org. TCGA, The Cancer Genome Atlas. **b** The expression levels of the predicted candidates in 778 breast cancer tissues and 87 adjacent normal tissues were obtained from TCGA database. **c** and **d** After transfecting MDA-MB-231 cells with miR-32-5p and miR-92b-3p mimics, the individual expression levels were examined using real-time PCR. N.C, scramble control. **e** and **f** The ectopic expression of miR-32-5p and miR-92b-3p suppressed the endogenous expression of mRNA and proteins. **g** and **h** Schema of the luciferase constructs. The miR-32-5p and miR-92b-3p targeting sequence in 3’UTR sequence of IDH1 is shown in the upper panel and the mutant (mut) of its seed region is shown in red, respectively. **i** and **j** Schema of the luciferase reporter constructs (upper panels). The relative luciferase activity of the reporter with wild-type and mutant 3′-UTR of IDH1 was determined in breast cancer cells co-transfected with miR-32-5p and miR-92b-3p mimic, respectively. The firefly luciferase activity was used as the normalization control

A number of studies have reported that dysfunction of miRNA is often the cause of the aberrant expression of cancer-associated genes [14, 16, 17]. Therefore, we further identified putative miRNAs that suppress IDH1 expression. Both microRNA.Org and TargetScan predicted 15 miRNA candidates to potentially target the 3′-UTR of IDH1 (Fig. 3a). Because the expression levels of IDH1 were lower in breast cancer, those miRNA candidates with elevated expression in breast cancer would have the potential to regulate IDH1. Therefore, the expression levels of the predicted candidates were examined using TCGA database, showing that miR-32-5p and miR-92b-3p expression was significantly higher (fold change > 2) in breast cancer tissues (n = 778) compared with adjacent normal tissues (n = 87; Fig. 3b). According to the aforementioned criteria, miR-32-5p and miR-92b-3p might suppress IDH1 expression by targeting its 3′-UTR. To examine this notion, miR-32-5p and miR-92b-3p were ectopically expressed in MDA-MB-231 cells by transfecting them with miR-32-5p and miR-92b-3p mimics. As shown in Fig. 3c and d, the expression levels of miR-32-5p and miR-92b-3p were clearly increased in MDA-MB-231 cells transfected with the respective mimics compared with those transfected with scramble control (N.C). Furthermore, the ectopic expression of miR-32-5p and miR-92b-3p suppressed the mRNA and protein expression of IDH1 in breast cancer cells (Fig. 3e and f). We further constructed the wild-type IDH1 full-length 3’UTR, the mutant IDH1 3’UTR with mutated miR-32-5p binding site and with mutated miR-92-3p binding site respectively into the pMIR reporter vector and performed luciferase reporter assays in MDA-MB-231 cells (Fig. 3g and h). As shown in Fig. 3i and j, miR-32-5p and miR-92b-3p significantly suppressed the luciferase activity of wild-type pMIR–IDH1-3′-UTR (wild-type), but not of mutated pMIR–IDH1-3′-UTR.

We examined the expression levels of miR-32-5p and miR-92b-3p in breast cancer using a real-time PCR approach. The expression levels of miR-32-5p and miR-92b-3p were significantly higher in breast cancer tissues than in the adjacent normal tissues (miR-32-5p, *p* < 0.001 and miR-92b-3p, *p* < 0.001, respectively; Additional file 5: Figure S1a and b). The ectopic expression of miR-32-5p or miR-92b-3p significantly accelerated the invasion ability of breast cancer cells (Additional file 5: Figure S1c and d). In summary, the aberrant overexpression of oncogenic miR-32-5p and miR-92b-3p resulted in the reduction of IDH1 by directly binding at the 3′-UTR of IDH1 to promote invasion in breast cancer cells.

### IDH1 expression contributed to breast cancer cell motility

**Fig. 4:**
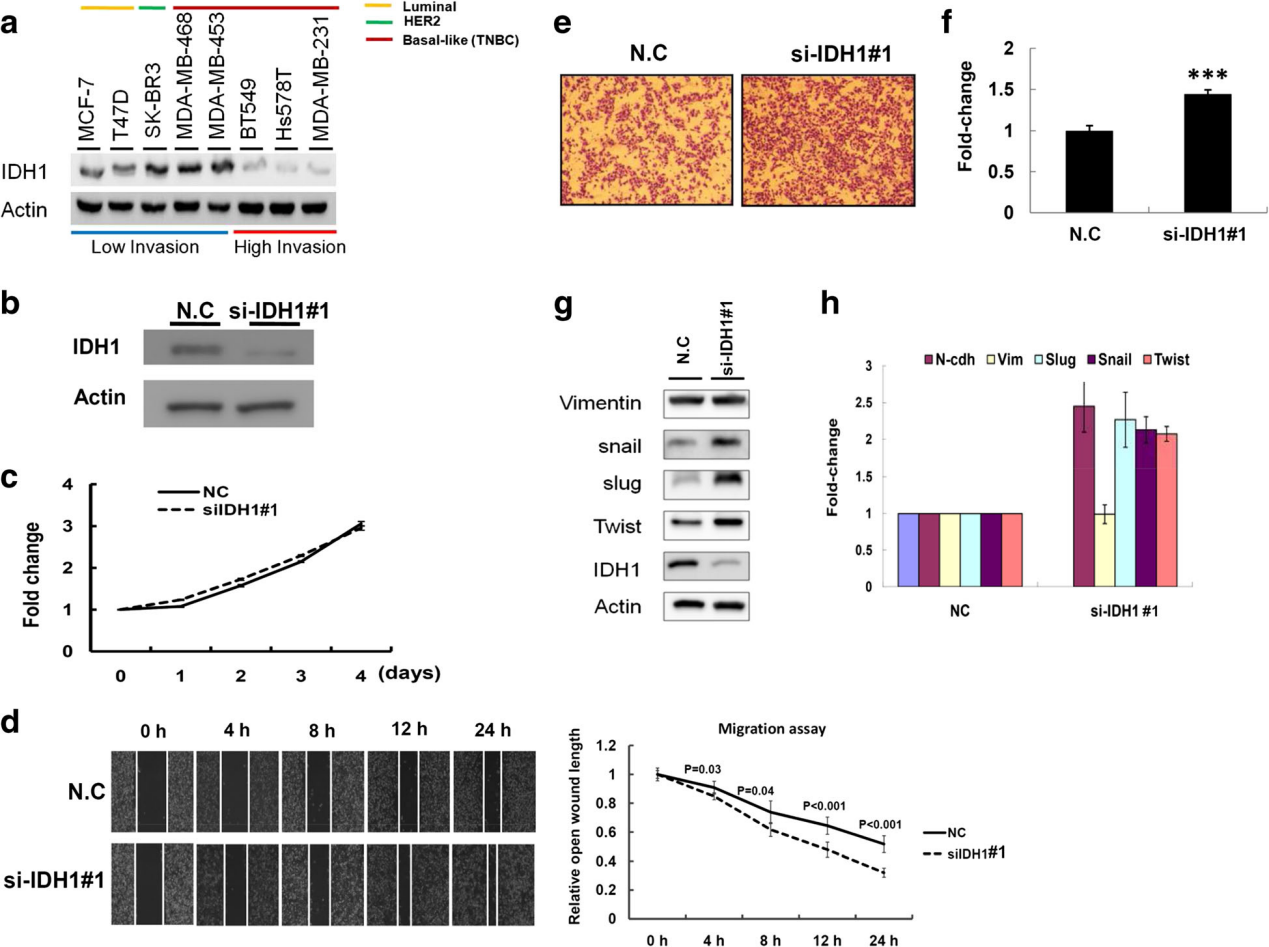
Isocitrate dehydrogenase 1 (IDH1) knockdown significantly promoted MDA-MB-231 cell motility. **a** Expression levels of IDH1 were examined in eight breast cancer cell lines: HER2, human epidermal growth factor receptor 2; TNBC, triple negative breast cancer. **b** After siRNA transfection with si-IDH1#1, the expression levels of IDH1 were examined in MDA-MB-231 cells through western blotting: N.C, scramble control. **c** Proliferation assay was performed in MDA-MB-231 cells transfected with scramble control and si-IDH1#1. **d** Wound healing assay was used in MDA-MB-231 cells transfected with si-IDH1#1 and scramble control. Relative migration ability was quantified by calculating the open wound length (right panel). **e** Invasion ability was assessed using the Transwell assay in MDA-MB-231 cells with si-IDH1#1 and scramble control. The cell images of a representative experiment are shown. **f** Values quantified using Ascent software are shown. Data are reported as the number of invading cells relative to the control (means ± standard deviation (S)). **g** and **h** Expression levels of epithelial–mesenchymal transition (EMT)-related markers were examined in MDA-MB-231 cells transfected with si-IDH1#1 and scramble control, through western blotting and real-time qPCR

Our data revealed that IDH1 expression levels were closely associated with early pathology and pN stages, suggesting that IDH1 contributes to the growth and migration/invasion of breast cancer cells. We further examined the expression levels of IDH1 in eight breast cancer cell lines, including cells with low invasive ability (MCF7, T47D, SK-BR3, MDA-MB-468, and MDA-MB-453) and cells with high invasion ability (BT549, Hs578T, and MDA-MB-231; Fig. 4a). Our data indicated that IDH1 expression levels were higher in cells with low invasive ability than in cells with high invasive ability. Endogenous IDH1 expression was knocked down in MDA-MB-231 cells through transfecting with si-IDH1 and scramble control. As shown in Fig. 4b and Additional file 6: Figure S2a, IDH1 expression levels were significantly lower in MDA-MB-231 cells transfected with si-IDH1 than in those transfected with scramble control. We further investigated the effects of IDH1 on the proliferation, migration, and invasion of MD-MB-231 cells. The growth ability of MDA-MB-231 cells was not significantly affected after IDH1 knockdown (Fig. 4c and Additional file 6: Figure S2b). However, the wound healing assay indicated that IDH1 knockdown significantly increased the migration ability of MDA-B-231 cells (Fig. 4d). Furthermore, the invasion ability of MDA-MB-231 cells significantly increased after IDH1 knockdown (*p* < 0.001; Fig. 4e and f and Additional file 6: Figure S2c and d). The expression levels of slug, snail, and twist proteins and mRNA were significantly increased, whereas those of vimentin were not significantly changed in MDA-MB-231 cells transfected with si-IDH1 (Fig. 4g and h and Additional file 6: Figure S2e). We also established an IDH1 stable knockdown with shIDH1 MDA-MB-231 cells. Our data revealed that a stable knockdown could accelerate the invasion ability of MDA-MB-231 through elevating snail and slug protein expression levels (Additional file 7: Figure S3). Similar results also were observed in HS578T and BT549 cells with IDH1 knockdown (Additional file 8: Figure S4). We also examined the role of IDH1 in ER-expressing MCF7 cells. As depicted in Additional file 9: Figure S5, the knockdown of IDH1 can significantly accelerate the migration ability of MCF7 cells through increased snail and twist expression (Additional file 9: Figure S5b and c). In contrast to MDA-MB-231 cells, the proliferation of MCF7 was promoted after IDH1 knockdown (Additional file 9: Figure S5d), which suggested that the role of IDH1 in MCF7 cell proliferation might be slightly different in breast cancer cells with different subtypes. However, these findings require further investigation. Our present study revealed that IDH1 knockdown significantly increased the migration and invasion ability of breast cancer cells by triggering their EMT.

### IDH1 regulated snail expression by modulating mitogen-activated protein kinase–HIF-1a and nuclear factor kappa B signaling

**Fig. 5:**
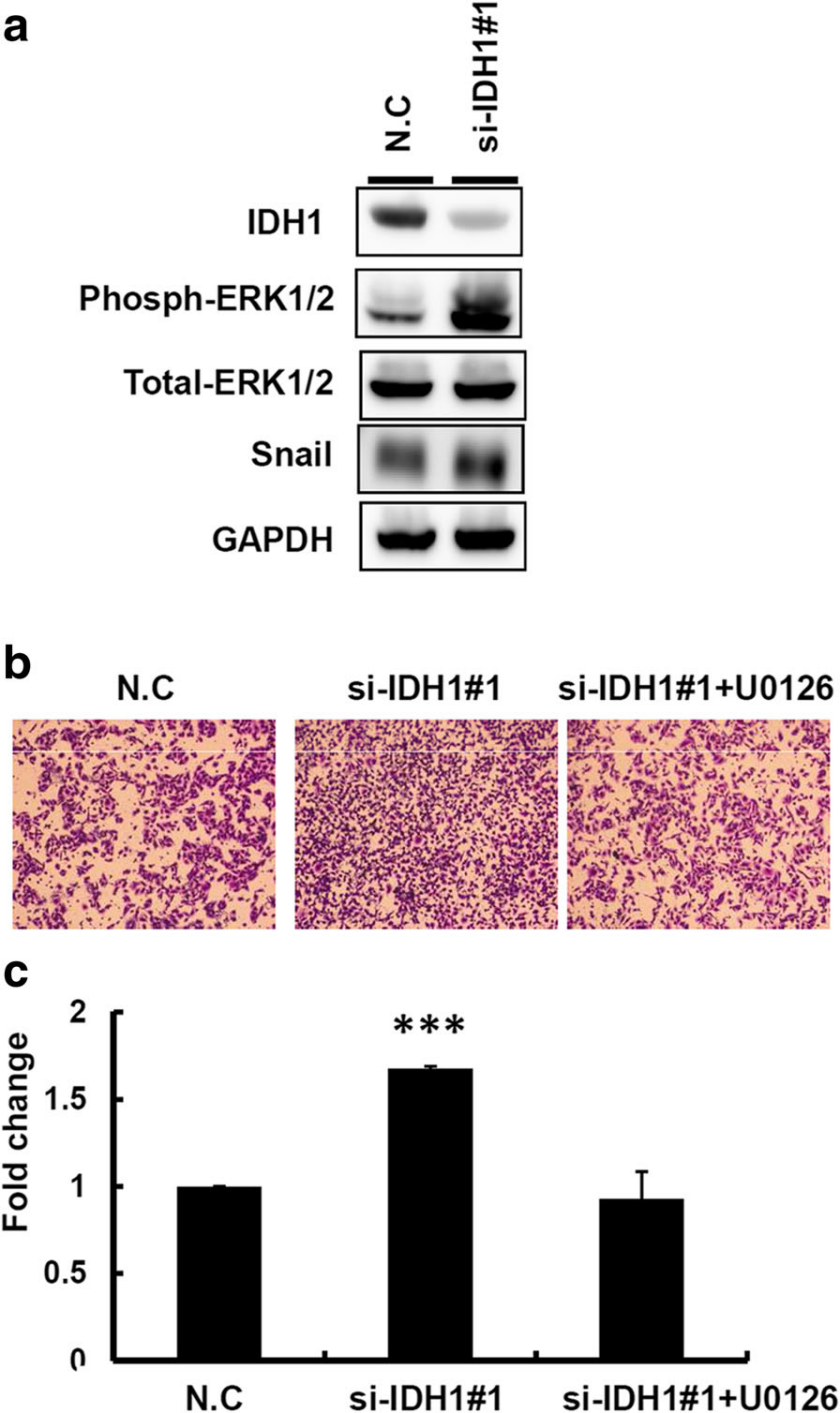
Isocitrate dehydrogenase 1 (IDH1) was involved in modulating breast cancer motility through mitogen-activated protein kinase (MAPK) signaling. **a** Western blot analysis of IDH1, p-extracellular signal-regulated kinase (ERK) 1/2, ERK1/2, snail, and glyceraldehyde-3-phosphate dehydrogenase (GAPDH) were present in MDA-MB-231 cells transfected with si-IDH1 and scramble control (N.C). **b** and **c** Cell invasion ability of IDH knockdown MDA-MB-231 cells with and without U0126 treatment. The relative invasion ability was further quantified using Ascent software

**Fig. 6:**
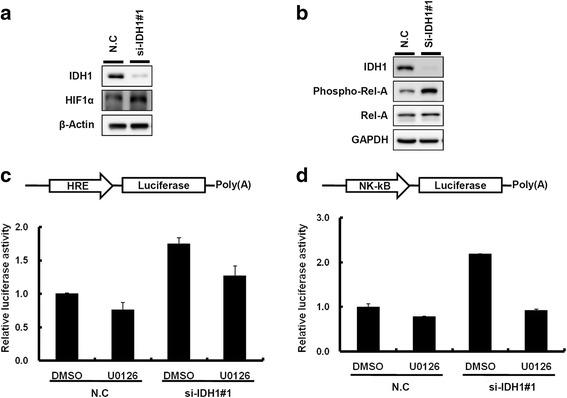
Isocitrate dehydrogenase 1 (IDH1) knockdown promoted breast cancer invasion through hypoxia-inducible factor-1 alpha (HIF-1α) and NFkB signaling. **a** and **b** Western blot analysis of IDH1, HIF-1α**,** NFkB, snail, and glyceraldehyde-3-phosphate dehydrogenase (GAPDH) are shown in MDA-MB-231 cells transfected with si-IDH1 and scramble control (N.C). **c** and **d** Schema of the luciferase reporter constructs (upper panels). The relative luciferase activity of HIF-1α and NFkB promoters were determined in breast cancer cells transfected with si-IDH1 and scramble control. Firefly luciferase activity was used as the normalization control. DMSO, dimethyl sulfoxide

We performed the transcriptome profiling of si-IDH1 and scramble control cells using a microarray approach to evaluate the putative signaling pathways regulated by IDH1. Pathway enrichment analysis revealed that IDH1 knockdown significantly altered cancer-related pathways such as the mitogen-activated protein kinase (MAPK), peroxisome proliferator-activated receptor signaling pathways, regulation of cell adhesion, and cell migration (Additional file 10: Figure S6). Mutant IDH1 can reportedly influence the activity of MAPK signaling in melanoma cells [18]. In addition, HIF-1a and nuclear factor kappa B (NFkB) are involved in EMT by elevating snail expression in cancer cells [19–23]. Based on the previous evidence, we examined whether IDH1 knockdown could potentially accelerate the invasion ability of breast cancer cells through MAPK–HIF-1a/NFkB signaling (Additional file 10: Figure S6b). As shown in Fig. 5a-c, MAPK signaling was activated in IDH1 knockdown MDA-MB-231 cells and thus promoted cell invasion. Treatment of U0126, the inhibitor of MAPK signaling, abolished IDH1 knockdown-induced cell invasion (Fig. 5b and c), implying that the effect of IDH1 knockdown on the promotion of cell invasion may be attributed to MAPK signaling. Further, the knockdown of IDH1 expression revealed that the expression levels of HIF-1α and phosphorylated NFkB were clearly elevated in MDA-MB-231 cells (Fig. 6a and b and Additional file 6: Figure S2e). The reporter assay showed that both HIF-1α and NFkB signaling were activated in IDH1 knockdown cells. Meanwhile, the activation of HIF-1α and NFkB signaling induced by IDH1 knockdown was abolished in the presence of U0126 (Fig. 6c and d). These results indicated that IDH1 possibly modulated the motility of breast cancer cells through MAPK–HIF-1a/NFkB signaling.

### IDH1 depletion increases the intracellular level of α-KG to promote HIF1α protein stability

**Fig. 7:**
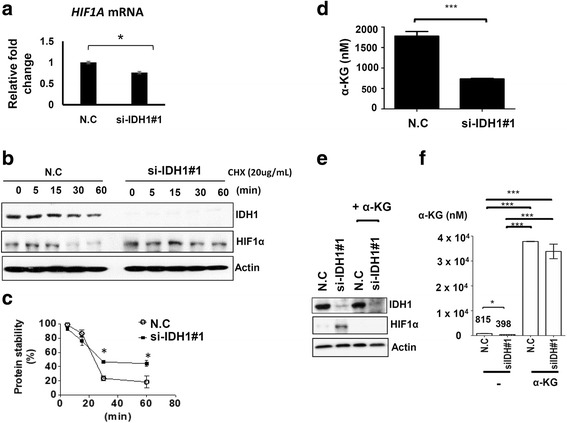
Depletion of isocitrate dehydrogenase 1 (IDH1) reduces the level of intracellular α-KG to stabilize hypoxia-inducible factor-1 alpha (HIF1α). **a** The effect of IDH1 depletion on *HIF1α* mRNA expression was analyzed through quantitative RT-PCR; *p < 0.05; N.C, scramble control. **b**, **c** The protein stability of HIF1α was examined in IDH1-depleted cells and control cells in the presence of 20 μg/mL of cycloheximide (CHX). Cells treated with CHX for the indicated time points were harvested for western blotting analysis; *p < 0.05. **d** Analysis of the intracellular level of α-KG in the control and IDH1-depleted cells using the UPLC system coupled with QT of MS. *** *p* < 0.001. **e** The effect of 1 mM α-KG treatment on HIF1α protein expression was analyzed through western blotting. **f** The intracellular level of α-KG in cells with the indicated treatments is shown; *p < 0.05, ***p < 0.001. Each experiment was performed in triplicate

We subsequently investigated the molecular mechanism underlying the IDH1 knockdown-mediated upregulation of HIF1α. First, we examined whether the elevated HIF1α expression upon IDH1 depletion was regulated at the mRNA level. Quantitative reverse transcription polymerase chain reaction (qRT-PCR) indicated that IDH1 depletion caused a slight decrease in *HIF1α* mRNA (Fig. 7a), which implied that the IDH1 depletion-induced upregulation of HIF1α may not be explained by the increased mRNA expression in *HIF1α*. Next, the effect of IDH1 depletion on the protein stability of HIF1α was examined. A protein degradation assay was performed to compare the protein stability of HIF1α between the IDH1-depleted cells and control cells. Western blotting indicated that the protein level of HIF1α was relatively stable during 60 min of cycloheximide treatment in the IDH1-depleted cells compared with the control cells, in which the protein level of HIF1α began to rapidly decrease at 30 min after cycloheximide treatment (Fig. 7b and c). This suggested that the IDH1 depletion-mediated upregulation of HIF1α was attributed to the increased protein stability of HIF1α. Several studies have demonstrated that the protein degradation of HIF1α is mainly regulated by an enzyme called prolyl hydroxylase domain-containing protein 2 (PHD2), the activity of which is highly dependent on α-KG under normoxic conditions [24, 25]. IDH1 is the major cellular enzyme responsible for catalyzing isocitrate into α-KG in the tricarboxylic acid (TCA) cycle [26], therefore, we hypothesized that IDH1 depletion caused a reduction in the cellular level of α-KG to impair the activity of PHD2, leading to the stabilization of HIF1α proteins under normoxic conditions. To address this assumption, the intracellular level of α-KG in IDH1-depleted cells was measured through UPLC coupled with a quadrupole time-of-flight mass spectrometer (QT of MS). The MS revealed that the intracellular level of α-KG decreased twofold in IDH1-depleted cells compared with the control cells (Fig. 7d). Furthermore, 1 mM α-KG reversed the increase in the HIF1α protein level observed in the IDH1-depleted cells (Fig. 7e and f). Taken together, these results indicated that IDH1 depletion resulted in a low level of cellular α-KG, which in turn impaired the activity of the α-KG-dependent enzyme PHD2, ultimately leading to the accumulation of HIF1α protein.

### IDH1^low^snail^high^ molecular signature is an independent marker for poor prognosis in breast cancer

**Fig. 8:**
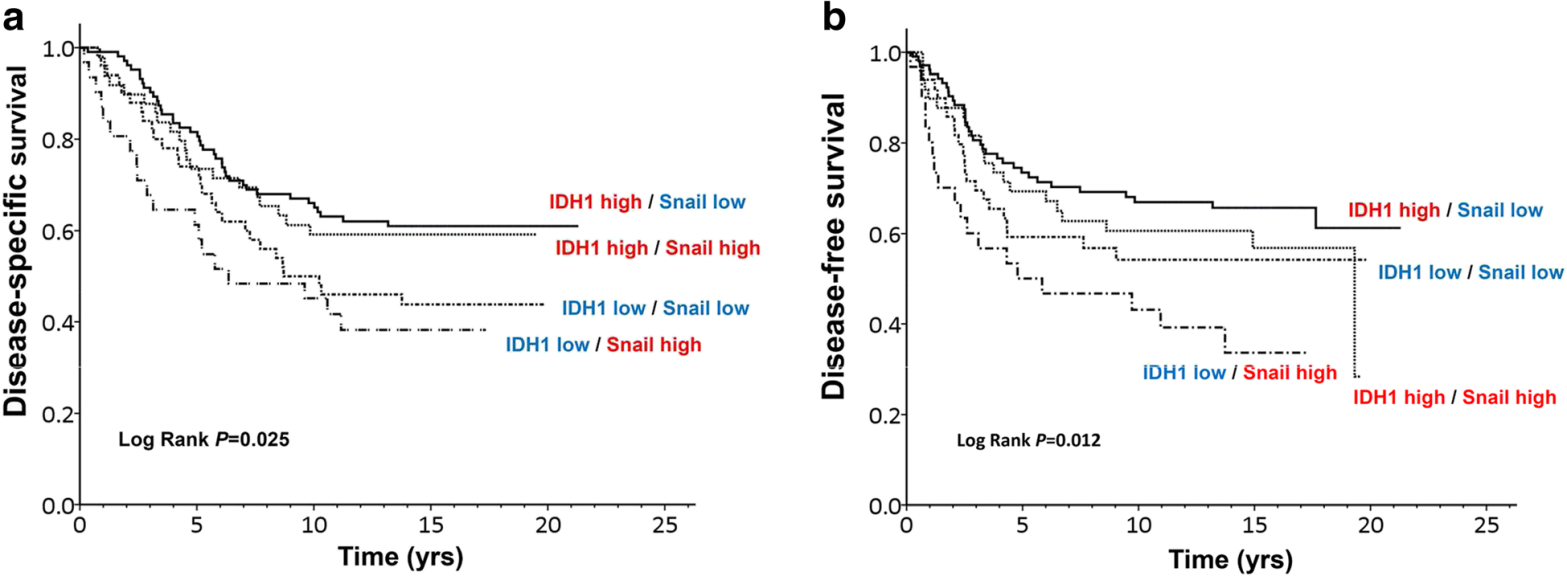
Combination of isocitrate dehydrogenase (IDH1) and snail expression levels closely correlated with the survival curve of patients with breast cancer. **a** and **b** Disease-specific survival and disease-free survival were compared according to IDH1 and snail expression in breast cancer cells using the log-rank test

Using the IHC data, we examined the correlation among IDH1, HIF-1a, twist, and snail. As shown in Table 3, the expression levels of HIF-1a and twist were positively associated with those of IDH1 (*r* = 0.221, *p* = 0.001 and *r* = 0.172, *p* = 0.011, respectively), whereas snail expression levels were inversely associated with IDH1 expression levels (*r* = − 0.141, *p* = 0.037). Notably, the IDH1–snail axis supported our finding in breast cancer on the negative correlation between IDH1 and snail expression in clinical samples. We next combined the expression levels of IDH1 and snail to assess the correlation with breast cancer survival. We classified the combined status of IDH1 and snail into four groups: IDH1 high–snail low, low levels of both IDH1 and snail, high levels of both IDH1 and snail, and IDH1 low–snail high. As shown in Fig. 8 and Table 4, low IDH1 expression levels and high snail expression levels were significantly associated with poor survival in patients with breast cancer (CHR for DSS, 2.10; 95% CI, 1.22–3.63; *p* = 0.008 and CHR for DFS, 2.49; 95% CI, 1.42–4.37, *p* = 0.001), even after adjustment for clinicopathological factors (AHR for DSS, 2.34; 95% CI, 1.32–4.16; *p* = 0.004 and AHR for DFS, 2.50; 95% CI, 1.39–4.50; *p* = 0.002). The combination of IDH1 and snail expression levels had a linear trend for their effects on DSS (*p* for linear trend = 0.006) and DFS (*p* for linear trend = 0.011).

**Table 3:** Correlation coefficients (r) for expression levels of four proteins in patients with breast IDC (n = 218)

Variables	IDH1	HIF1α	Snail	Twist
IDH1	-	-	-	-
	-	-	-	-
HIF1α	***r*** **=** ***0.221***	-	-	
	***p*** **=** ***0.001***	-	-	
Snail	***r*** **= −** ***0.141***	***r*** **=** ***0.194***	-	
	***p*** **=** ***0.037***	***p*** **=** ***0.004***	-	
Twist	***r*** **=** ***0.172***	***r*** **=** ***0.424***	*r* = 0.115	-
	***p*** **=** ***0.011***	***p*** **<** ***0.001***	*p* = 0.090	-

The correlation coefficients and *p* values were estimated by Spearman’s rank correlation

*IDC* invasive ductal carcinoma, *IDH1* isocitrate dehydrogenase 1, *HIF1α* hypoxia-inducible factor-1 alpha

Bold italic values denote statistically significant

**Table 4:** Univariate and multivariate Cox’s regression analysis of the combined IDH1 and Snail for disease-specific survival and disease-free survival of breast IDC patients

Variable (n = 234)	Number (percentage)	Disease-specific survival				Disease-free survival			
		CHR^*^ (95% CI)	*p value*	AHR^**^ (95% CI)	*p value*	CHR^*^ (95% CI)	*p value*	AHR^**^ (95% CI)	*p value*
IDH1 high, snail low	104 (44.4)	1.00		1.00		1.00		1.00	
IDH1 high, snail high	49 (20.9)	1.10 (0.64–1.88)	0.725	1.15 (0.67–1.97)	0.624	1.30 (0.75–2.23)	0.349	1.31 (0.76–2.27)	0.331
IDH1 low, snail low	50 (21.4)	1.63 (1.01–2.64)	***0.047***	1.44 (0.88–2.36)	0.151	1.49 (0.88–2.54)	0.142	1.23 (0.72–2.13)	0.450
IDH1 low, snail high	31 (13.3)	***2.10*** **(** ***1.22*** **–** ***3.63*** **)**	***0.008***	***2.34*** **(** ***1.32*** **–** ***4.16*** **)**	***0.004***	***2.49*** **(** ***1.42*** **–** ***4.37*** **)**	***0.001***	***2.50*** **(** ***1.39–4.50*** **)**	***0.002***
		*p* for linear trend	***0.004***	*p* for linear trend	**0.006**	*p* for linear trend	***0.002***	*p* for linear trend	**0.011**

*Abbreviations*: *IDH1* isocitrate dehydrogenase 1, *IDC* invasive ductal carcinoma, *CHR* crude hazard ratio, *AHR* adjusted hazard ratio

*CHRs were estimated by univariate Cox regression

**AHRs were adjusted for American Joint Committee on Cancer pathological stage (II and III vs. I), grading (III vs. I and II), incomplete or inappropriate adjuvant treatment vs. non-treatment or complete adjuvant treatment, and molecular subtypes (luminal B, human epidermal growth factor receptor 2 (Her2) overexpression and basal-like vs. luminal A) by multivariate Cox regression

Bold italic values denote statistically significant

**Fig. 9:**
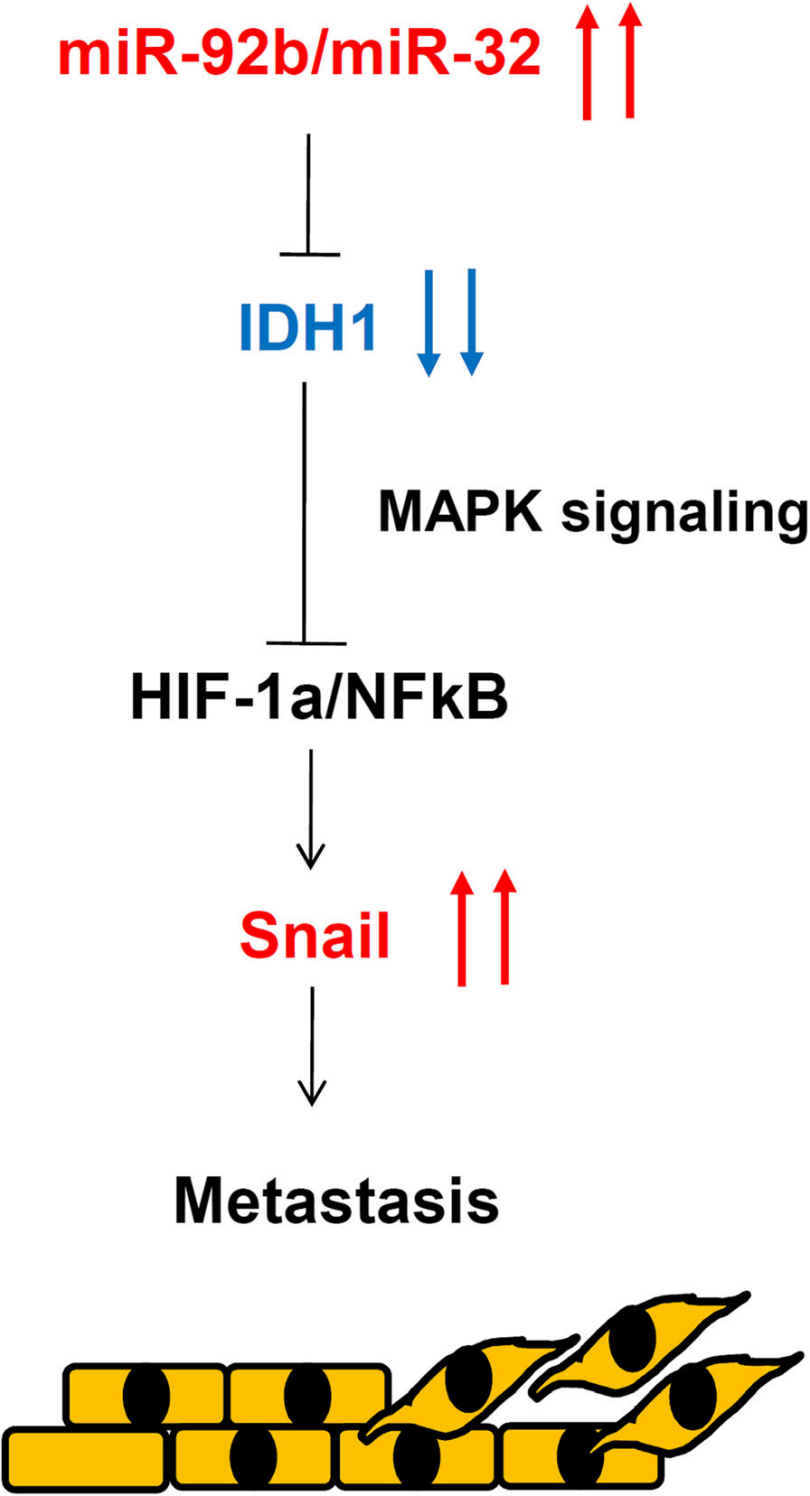
Model for determining the role of isocitrate dehydrogenase 1 (IDH1) in modulating the motility of breast cancer cells; MAPK, mitogen-activated protein kinase

In summary, we observed that miR-32-5p and miR-92b-3p overexpression resulted in tumor-suppressive IDH1 silencing in breast cancer tissues compared with that in adjacent normal tissues. Furthermore, depletion of IDH1 in breast cancer cells results in accelerating breast cancer migration and invasion activities by activating snail expression (Fig. 9). Low IDH1 expression levels, particularly in combination with snail expression, served as a potential prognosis biomarker for breast cancer.

## Discussion

There are reports from studies that IDH genes play crucial roles in the metabolism of glucose, fatty acids, and glutamine in humans [6, 7]. NADPH is an essential reducing agent for glutathione (GSH) regeneration by GSH reductase and the NADPH-dependent thioredoxin system, both of which are important for protecting cells from oxidative damage. IDH1 and IDH2 can produce cytosolic NADPH in cells [27], suggesting that they defend against the oxidative stress caused by reactive oxygen species [27, 28]. Therefore, the disruption of the normal functions of IDH1 and IDH2 may significantly affect the cellular redox balance and result in cancer cell growth dysfunction [28–31]. Studies have revealed that IDH1 and IDH2 mutations promote global hypermethylation with concomitant reductions in 5hmC levels [32–34]. In previous studies, IDH mutations were rarely detected in human solid tumors, except for glioma [8, 11, 35]. Therefore, IDH repression seems to be a more crucial mechanism for regulating global DNA methylation in solid tumors, rather than IDH mutations, which are more common in gliomas and hematological malignancies [18].

In our previous work, we reported that the contents of 5mC and 5hmC were significantly lower in breast cancer as compared to those in corresponding adjacent normal tissues [13]. We also revealed that IDH1 and IDH2 modulate 5hmC levels in human gastric cancer [36]. However, only IDH2 expression levels were significantly lower in gastric cancer tissues than in adjacent normal tissues. Furthermore, low IDH2 expression levels have been closely associated with the poor survival curve in patients with gastric cancer [36]. In this study, we provided a novel insight to clarify the role of IDH1 expression in breast cancer. Our results indicated that IDH1 expression levels were significantly lower in breast cancer tissues than in adjacent normal tissues and that IDH1 played a crucial role in modulating breast cancer metastasis. Furthermore, low IDH1 expression was significantly associated with the poor prognosis in breast cancer. A study has shown that the siRNA knockdown of either IDH1 or IDH2 can significantly reduce the proliferative capacity of a glioblastoma cell line expressing both wild-type IDH1 and IDH2 [30]. Lee et al. suggested that the attenuation of IDH family gene expression may protect the skin from ultraviolet (UV)-B-mediated damage by inducing the apoptosis of UV-damaged cells [31]. According to these findings, IDH genes seem to play a crucial role in cell growth by maintaining a normal cellular redox status. Although we observed that the low expression levels of IDH1 in IDC were associated with poor clinical pathological features of breast cancer, IDH1 depletion showed no significant effects on the growth of breast cancer cells (Fig. 4b). Furthermore, the siRNA knockdown of IDH1 can promote the invasion ability of breast cancer cells, suggesting that IDH1 acts as a tumor suppressor in breast cancer progression. Wang et al. reported that the expression levels of IDH1 and IDH2 were significantly decreased in breast cancer cells with adriamycin resistance, implying that IDH acts as a tumor suppressor in breast cancer drug resistance [37]. Altogether, these results implied that the IDH family possibly has distinct biological functions in human cancer.

We first reported that the loss of IDH1 expression resulted from the aberrant overexpression of miR-32-5p and miR-92b-3p in breast cancer. Studies have indicated that miR-32-5p plays an oncogenic role in the growth and invasion ability of breast cancer cells by directly silencing PHLPP2 and FBXW7 [38, 39]. On the other hand, the biological role of miR-92b-3p is controversial and unknown in human breast cancer. miR-92b-3p has been reported to play a tumor-suppressing role in esophageal cancer by silencing RAB23 or integrin α6 [40, 41]. Contrastingly, miR-92b-3p has also been reported to play an oncogenic role in the growth and invasion ability by regulating phosphatase and tensin homolog and Smad3 in bladder cancer, hepatocellular carcinoma, and glioblastoma cells [42–44]. Herein, we reported a novel finding that the expression levels of miR-92b-3p significantly increased and promoted breast cancer invasion by silencing the expression of IDH1.

IDH1 mutants can play a dominant negative role in wild-type IDH1 functions, resulting in the increased phosphorylation of MAPK and signal transducer and activator of transcription 3 in melanoma cells [18]. Zhao et al. reported that IDH1 mutants can activate the hypoxia pathway by preventing HIF-1α degradation through prolyl hydroxylase activation in glioma cells [9]. Studies have revealed that HIF-1α can promote cancer cell metastasis through EMT by upregulating snail, twist, and vimentin expression [21–23]. In the present study, we demonstrated that IDH1 depletion in breast cancer cells led to an increase in the HIF1α protein level to promote cell migration and invasion. An α-KG-dependent dioxygenase called prolyl hydroxylase 2 (PHD2) regulates the protein stability of HIF1α under normoxic conditions by hydroxylating the proline residues 402 and 405 on the oxygen-dependent degradation domain at its N terminus [24], and hydroxylated HIF1α is subjected to protein degradation by the 26S proteasome [45]. Our mechanistic study demonstrated that IDH1 depletion led to a decrease in intracellular α-KG, which in turn stabilized HIF1α (Fig. 7). The addition of 1 mM α-KG reversed the increase of HIF1α in IDH1-depleted cells. Furthermore, we also demonstrated that the depletion of IDH1 did not increase the mRNA level of HIF1α. Therefore, we reasoned that IDH1 depletion caused the low level of intracellular α-KG to impair PHD2 enzyme activity, which stabilized HIF1α. Collectively, these results demonstrated that IDH1 plays a tumor-suppressing role in the progression of breast cancer.

## Conclusion

We provide a novel insight that miR-32-5p and miR-92b-3p dysregulation results in IDH1 depletion. Low IDH1 expression levels can promote the migration and invasion abilities of breast cancer cells by activating snail expression. IDH1^low^snail^high^ molecular signature might serve as a favorable independent marker for poor breast cancer survival.

## Additional files

Additional file 1: **Table S1.** Primer sequence list. (DOC 38 kb)

Additional file 2: **Table S2.** Antibody information. (DOC 32 kb)

Additional file 3: **Table S3.** Correlation of IDH1 expression with molecular markers in patients with breast cancer. (DOCX 16 kb)

Additional file 4: **Table S4.** Univariate Cox regression analysis of IDH1 expression and overall survival in 1070 patients with breast cancer from TCGA database. (DOC 30 kb)

Additional file 5: **Figure S1.** Oncogenic miRNAs, miR-32-5p, and miR-92b-3p accelerated breast cancer cell invasion. (**a**) and (**b**) Expression levels of miR-32-5p and miR-92b-3p were assessed in breast cancer tissues (n = 51) and adjacent normal tissues (n = 29) by using a real-time PCR approach. (**c**) Invasion ability was assessed using the Transwell assay in MDA-MB-231 cells transfected with miR-32-5p, miR-92b-3p, and scramble control. The cell images of a representative experiment are shown. (**d**) Values quantified using Ascent software. Data are reported as the number of colonies relative to the control (means ± SD). (TIFF 3710 kb)

Additional file 6: **Figure S2.** IDH1 knockdown significantly promoted MDA-MB-231 cell motility. (**a**) After siRNA transfection with siIDH1#2, the expression levels of IDH1 were examined in MDA-MB-231 cells through western blotting. (**b**) A proliferation assay was performed in MDA-MB-231 cells transfected with the scrambled control and si-IDH1#2. (**c**) Invasion ability was assessed using the Transwell assay in MDA-MB-231 cells with si-IDH1#2 and the scrambled control. The cell images of a representative experiment are provided. (**d**) Values quantified using Ascent software. Data are reported as the number of invading cell relative to the control cells (means ± standard deviation (SD)). (**e**) Expression levels of IDH1, snail, slug, twist and actin were examined in MDA-MB-231 cells transfected with si-IDH1#2 and the scrambled control through western blotting. (TIFF 2799 kb)

Additional file 7: **Figure S3.** IDH1 stable knockdown significantly promoted MDA-MB-231 cell motility. (**a**) The expression levels of IDH1 were examined in two IDH1 stable knockdown MDA-MB-231 cells (shIDH1#1 and shIDH1#2) through western blotting. (**b**) Invasion ability was assessed using the Transwell assay in MDA-MB-231 cells with IDH1 stable knockdown and a scrambled control. The cell images of the representative experiment are provided. (**c**) Values were quantified using Ascent software, as detailed. Data are reported as the number of invading cells relative to the control (means ± standard deviation (SD)). (**d**) Expression levels of IDH1, snail, slug, twist, and actin were examined in shIDH1#1, shIDH1#2, and the scrambled control through western blotting. (TIFF 3572 kb)

Additional file 8: **Figure S4.** IDH1 knockdown significantly accelerated HS578T and BT549 cell motility. (**a**), (**d**) Invasion ability was assessed using the Transwell assay in HS578T and BT549 cells with si-IDH1 and scramble control. The cell images of a representative experiment are shown. (**b**), (**e**) Values quantified using Ascent software are shown. Data are reported as the number of colonies relative to the control (means ± standard deviation (SD)). (**c**), (**f**) Expression levels of EMT-related markers were examined in HS578T and BT549 cells transfected with si-IDH1 and scramble control by western blotting. (TIFF 4403 kb)

Additional file 9: **Figure S5.** IDH1 knockdown accelerated MCF7 cell proliferation and migration ability. (**a**) the expression levels of IDH1 were examined in MCF7 cells with siIDH1#1, siIDH1#2, and control transfection through western blotting. (**b**) A wound healing assay was employed to examine MCF7 cells transfected with siIDH1#1, siIDH1#2, and the scrambled control. (**c**) The expression levels of IDH1, snail, slug, twist and actin were examined in siIDH1#1, siIDH1#2, and the scrambled control through western blotting. (**d**) The proliferation assay was performed in MCF-7 cells transfected with the scrambled control, siIDH1#1 and siIDH1#2, respectively. (TIFF 4527 kb)

Additional file 10: **Figure S6.** Gene set enrichment analysis for differential expression of genes in MDA-MB-231 cells transfected with si-IDH1 compared with those transfected with scramble control. (**a**) Differential expression of genes (upregulated or downregulated twofold change) was identified using a microarray approach. These gene sets were significantly enriched in metastasis-associated terms from the Kyoto Encyclopedia of Genes and Genomes. (**b**) Schematic putative signaling pathway illustrating IDH1-modulated cancer cell invasion. (TIFF 1728 kb)

## Abbreviations

ΔCt: Difference in cycle threshold
AHR: Adjusted hazard ratio
ANOVA: Analysis of variance
CHR: Crude hazard ratio
CI: Confidence interval
DCIS: Ductal carcinoma in situ
DFS: Disease-free survival
DSS: Disease-specific survival
EMT: Epithelial–mesenchymal transition
FBS: Fetal bovine serum
HIFα: Hypoxia-inducible factor-1 alpha
IDC: Invasive ductal carcinoma
IDH: Isocitrate dehydrogenase
IHC: Immunohistochemistry assay
IRB: Institutional Review Board
MAPK: Mitogen-activated protein kinase
miRNA: Micro RNA
NADPH: Nicotinamide adenine dinucleotide phosphate-oxidase
NFκB: Nuclear factor kappa B
PHD2: Prolyl hydroxylase 2
RIPA: Radioimmunoprecipitation assay
shRNA: Short hairpin RNA
TCGA: The Cancer Genome Atlas
UTR: Untranslated region
