# Supplementary material for: Isocitrate dehydrogenase 1–snail axis dysfunction significantly correlates with breast cancer prognosis and regulates cell invasion ability
Source: Breast Cancer Res. 2018 Apr 16;20:25. doi: 10.1186/s13058-018-0953-7 (PMC5902927; doi:10.1186/s13058-018-0953-7)
Supplement: Supplementary file 1 — Table S1. Primer sequence list. (DOC 38 kb) [file 13058_2018_953_MOESM1_ESM.doc]

| **Table S1. Primers sequence list** |  |
| --- | --- |
| **protein-coding genes** | **primer sequences** |
| GAPDH-F | TGCACCACCAACTGCTTAGC |
| GAPDH-R | GGCATGGACTGTGGTCATGAG |
| IDH1-F | TGCAAAAATATCCCCCGGCT |
| IDH2-R | TACATCCCCATGGCAACACC |
| Twist-F | GCCAATCAGCCACTGAAAGG |
| Twist-R | TGTTCTTATAGTTCCTCTGATTGTTACCA |
| E-cad-F | GTCACTGACACCAACGATAATCCT |
| E-cad-R | TTTCAGTGTGGTGATTACGACGTTA |
| Snail-F | ACCACTATGCCGCGCTCTT |
| Snail-R | GGTCGTAGGGCTGCTGGAA |
| Slug-F | TGTTGCAGTGAGGGCAAGAA |
| Slug-R | GACCCTGGTTGCTTCAAGGA |
| Vimentin-F | CCAAACTTTTCCTCCCTGAACC |
| Vimentin-R | GTGATGCTGAGAAGTTTCGTTGA |
| HIF1a-F | GGTTCTCACAGATGATGGTG |
| HIF1a-R | TTCTTCCTCGGCTAGTTAGG |
| **microRNA** | **primer sequences** |
| U6-F | CTCGCTTCGGCAGCACA |
| U6-R | AACGCTTCACGAATTTGCGT |
| miR-32-5p-RT | CTCAACTGGTGTCGTGGAGTCGGCAATTCAGTTGAGTGCAACTT |
| miR-32-5p-GSF | CGGCGGTATTGCACATTACTAA |
| miR-92b-3p-RT | CTCAACTGGTGTCGTGGAGTCGGCAATTCAGTTGAGGGAGGCCG |
| miR-92d-3p-GSF | CGGCGGTATTGCACTCGTCCCG |
| Universal-R | CTGGTGTCGTGGAGTCGGCAATTC |
