# Supplementary material for: Isocitrate dehydrogenase 1–snail axis dysfunction significantly correlates with breast cancer prognosis and regulates cell invasion ability
Source: Breast Cancer Res. 2018 Apr 16;20:25. doi: 10.1186/s13058-018-0953-7 (PMC5902927; doi:10.1186/s13058-018-0953-7)
Supplement: Supplementary file 2 — Table S2. Antibody information. (DOC 32 kb) [file 13058_2018_953_MOESM2_ESM.doc]

| **Table S2. The antibody information** | | | |
| --- | --- | --- | --- |
| **antibody** | **dilution** | **Commpany** | **Host** |
| **anti-IDH1** | 1:2000 | GeneTex | Rabbit |
| **anti-IDH2** | 1:2000 | GeneTex | Rabbit |
| **anti-snail** | 1:2000 | Cell signaling | Rabbit |
| **anti-Twist** | 1:2000 | GeneTex | Rabbit |
| **anti-slug** | 1:100 | SANTA CRUZ | Mouse |
| **anti-Vimentin** | 1:2000 | GeneTex | Rabbit |
| **anti-RelA** | 1:2000 | Cell signaling | Rabbit |
| **anti-RelA-p** | 1:2000 | Cell signaling | Rabbit |
| **anti-HIF-1a** | 1:500 | GeneTex | Mouse |
