# Supplementary material for: Isocitrate dehydrogenase 1–snail axis dysfunction significantly correlates with breast cancer prognosis and regulates cell invasion ability
Source: Breast Cancer Res. 2018 Apr 16;20:25. doi: 10.1186/s13058-018-0953-7 (PMC5902927; doi:10.1186/s13058-018-0953-7)
Supplement: Supplementary file 3 — Table S3. Correlation of IDH1 expression with molecular markers in patients with breast cancer. (DOCX 16 kb) [file 13058_2018_953_MOESM3_ESM.docx]

| Table S3. Correlation of IDH1 expression with molecular marker of breast cancer patients | | | | |
| --- | --- | --- | --- | --- |
| Variable | IDH1 | | | |
|  | n | Mean±SD | Median | p-value |
|  | % |  |  |  |
| ER status | (n=252) |  |  |  |
| Negative | 38.9 | 4.82±2.01 | 6.00 | 0.462 |
| Positive | 61.1 | 4.63±1.92 | 5.00 |  |
| PgR status | (n=264) |  |  |  |
| Negative | 45.1 | 4.59±2.11 | 5.00 | 0.504 |
| Positive | 54.9 | 4.75±1.86 | 5.00 |  |
| Her2 status | (n=264) |  |  |  |
| Negative | 74.2 | 4.54±2.04 | 5.00 | **0.046** |
| Positive | 25.8 | 5.09±1.73 | 6.00 |  |
| ^a^p-value were estimated by student T test | | | | |
