# Supplementary material for: Isocitrate dehydrogenase 1–snail axis dysfunction significantly correlates with breast cancer prognosis and regulates cell invasion ability
Source: Breast Cancer Res. 2018 Apr 16;20:25. doi: 10.1186/s13058-018-0953-7 (PMC5902927; doi:10.1186/s13058-018-0953-7)
Supplement: Supplementary file 4 — Table S4. Univariate Cox regression analysis of IDH1 expression and overall survival in 1070 patients with breast cancer from TCGA database. (DOC 30 kb) [file 13058_2018_953_MOESM4_ESM.doc]

| **Table S4. Univariate Cox’s regression analysis of IDH1 expression for overall survival of 1070 patients with breast cancer from TCGA database.** | | | |
| --- | --- | --- | --- |
| **Characteristic** | **No. (%)** | **OS** | |
|  |  | **CHR (95% CI)** | **P-value** |
| **IDH1** | (n=1070) |  |  |
| High | 329 (30.7) | 1.00 |  |
| Low | 741 (69.3) | 1.80 (1.10 -2.94) | **0.020** |
|  |  |  |  |
| *CHR, crude hazard ratio* | | | |
