# Supplementary figures and images for: Isocitrate dehydrogenase 1–snail axis dysfunction significantly correlates with breast cancer prognosis and regulates cell invasion ability
Source: Breast Cancer Res. 2018 Apr 16;20:25. doi: 10.1186/s13058-018-0953-7 (PMC5902927; doi:10.1186/s13058-018-0953-7)

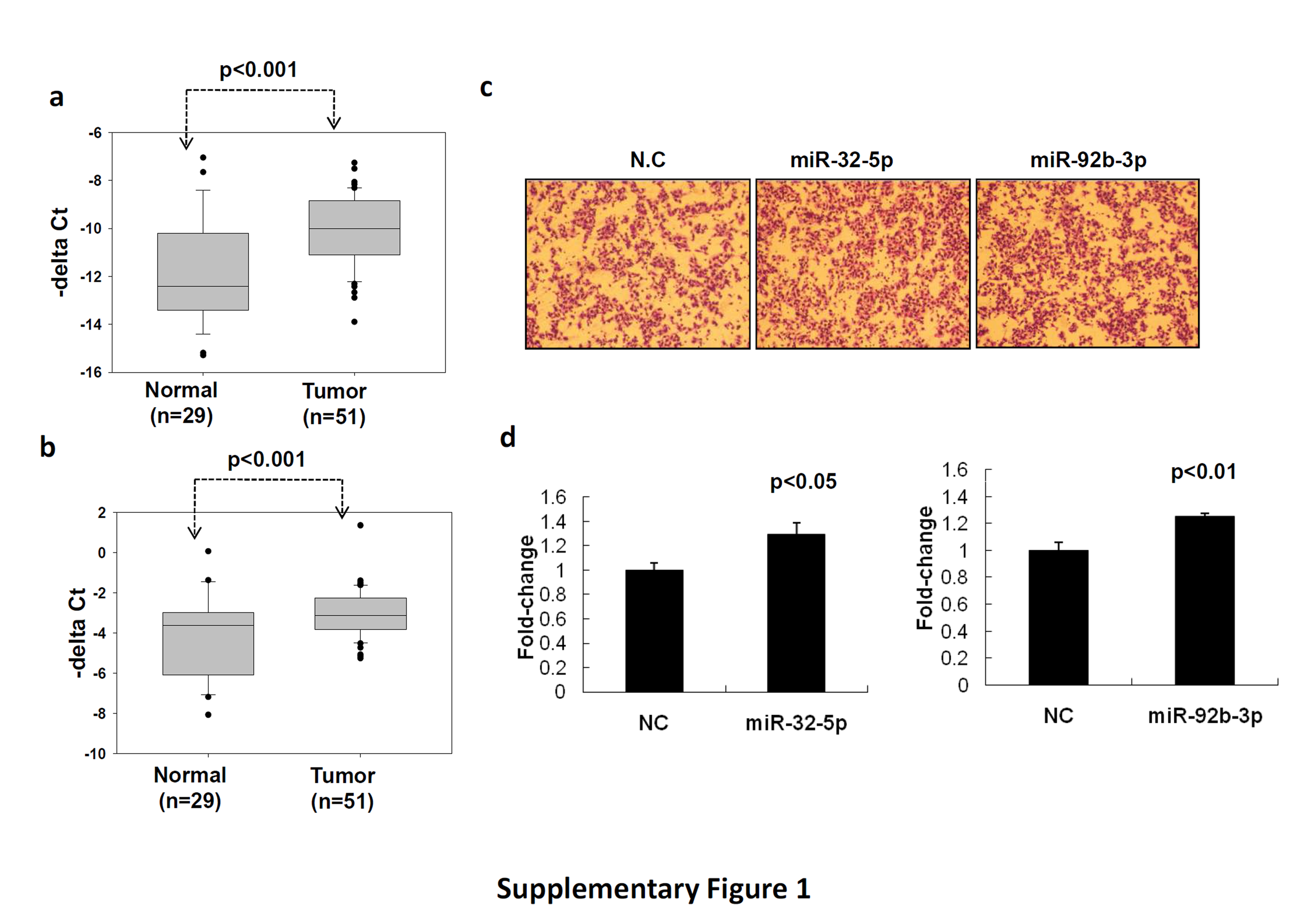

Supplement: Supplementary file 5 — Figure S1. Oncogenic miRNAs, miR-32-5p, and miR-92b-3p accelerated breast cancer cell invasion. (a) and (b) Expression levels of miR-32-5p and miR-92b-3p were assessed in breast cancer tissues (n = 51) and adjacent normal tissues (n = 29) by using a real-time PCR approach. (c) Invasion ability was assessed using the Transwell assay in MDA-MB-231 cells transfected with miR-32-5p, miR-92b-3p, and scramble control. The cell images of a representative experiment are shown. (d) Values quantified using Ascent software. Data are reported as the number of colonies relative to the control (means ± SD). (TIFF 3710 kb) [file 13058_2018_953_MOESM5_ESM.tif]

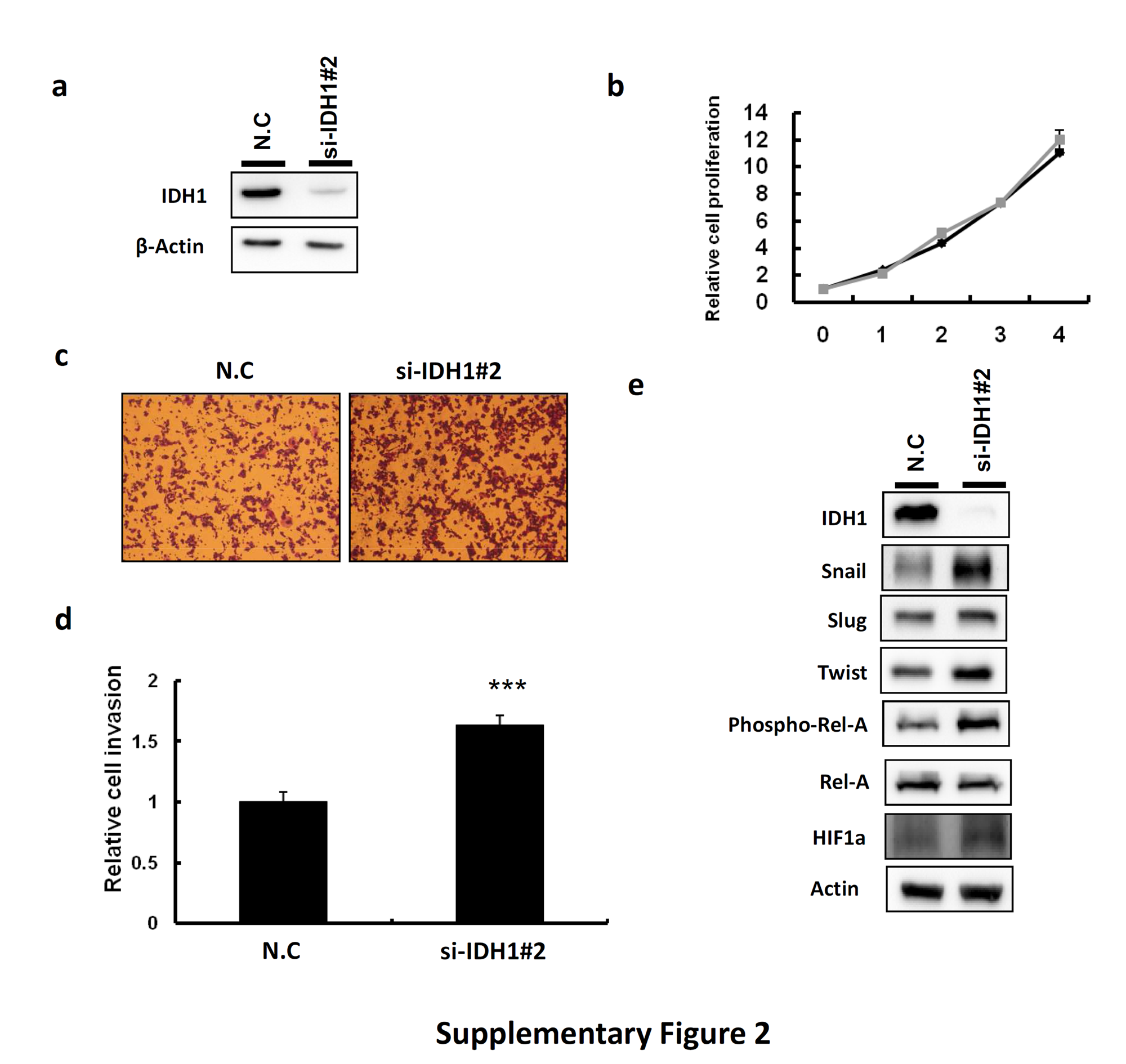

Supplement: Supplementary file 6 — Figure S2. IDH1 knockdown significantly promoted MDA-MB-231 cell motility. (a) After siRNA transfection with siIDH1#2, the expression levels of IDH1 were examined in MDA-MB-231 cells through western blotting. (b) A proliferation assay was performed in MDA-MB-231 cells transfected with the scrambled control and si-IDH1#2. (c) Invasion ability was assessed using the Transwell assay in MDA-MB-231 cells with si-IDH1#2 and the scrambled control. The cell images of a representative experiment are provided. (d) Values quantified using Ascent software. Data are reported as the number of invading cell relative to the control cells (means ± standard deviation (SD)). (e) Expression levels of IDH1, snail, slug, twist and actin were examined in MDA-MB-231 cells transfected with si-IDH1#2 and the scrambled control through western blotting. (TIFF 2799 kb) [file 13058_2018_953_MOESM6_ESM.tif]

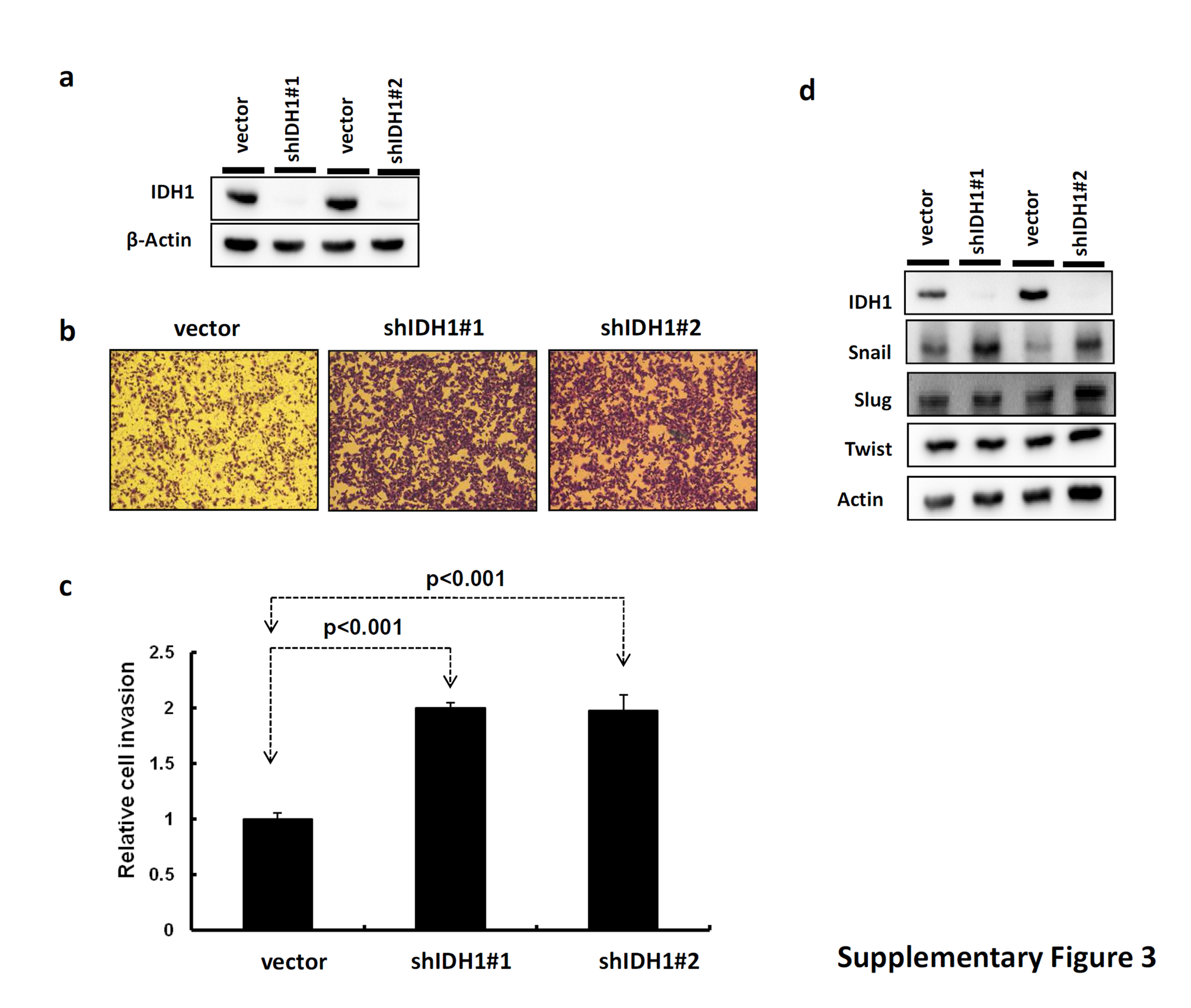

Supplement: Supplementary file 7 — Figure S3. IDH1 stable knockdown significantly promoted MDA-MB-231 cell motility. (a) The expression levels of IDH1 were examined in two IDH1 stable knockdown MDA-MB-231 cells (shIDH1#1 and shIDH1#2) through western blotting. (b) Invasion ability was assessed using the Transwell assay in MDA-MB-231 cells with IDH1 stable knockdown and a scrambled control. The cell images of the representative experiment are provided. (c) Values were quantified using Ascent software, as detailed. Data are reported as the number of invading cells relative to the control (means ± standard deviation (SD)). (d) Expression levels of IDH1, snail, slug, twist, and actin were examined in shIDH1#1, shIDH1#2, and the scrambled control through western blotting. (TIFF 3572 kb) [file 13058_2018_953_MOESM7_ESM.tif]

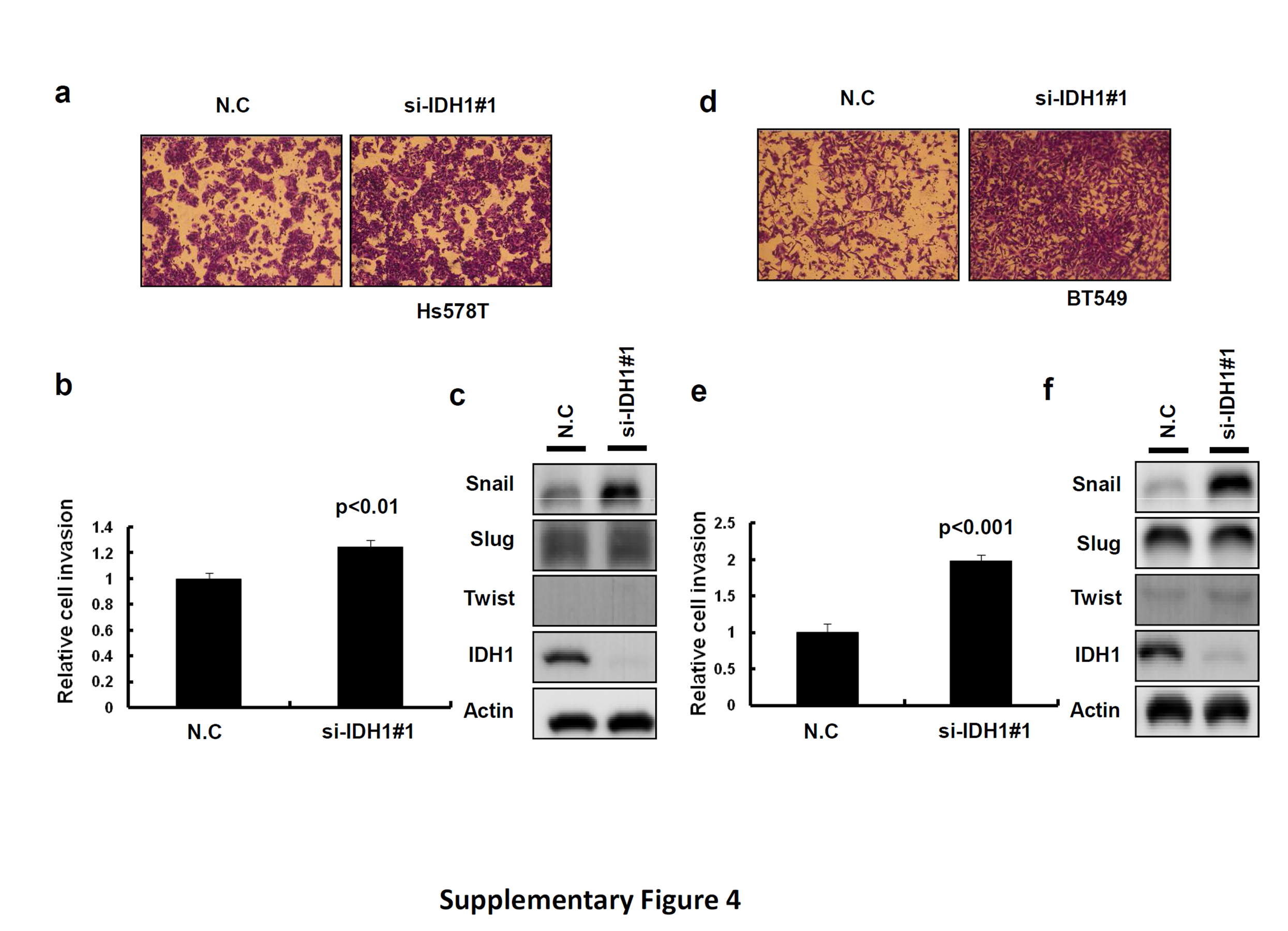

Supplement: Supplementary file 8 — Figure S4. IDH1 knockdown significantly accelerated HS578T and BT549 cell motility. (a), (d) Invasion ability was assessed using the Transwell assay in HS578T and BT549 cells with si-IDH1 and scramble control. The cell images of a representative experiment are shown. (b), (e) Values quantified using Ascent software are shown. Data are reported as the number of colonies relative to the control (means ± standard deviation (SD)). (c), (f) Expression levels of EMT-related markers were examined in HS578T and BT549 cells transfected with si-IDH1 and scramble control by western blotting. (TIFF 4403 kb) [file 13058_2018_953_MOESM8_ESM.tif]

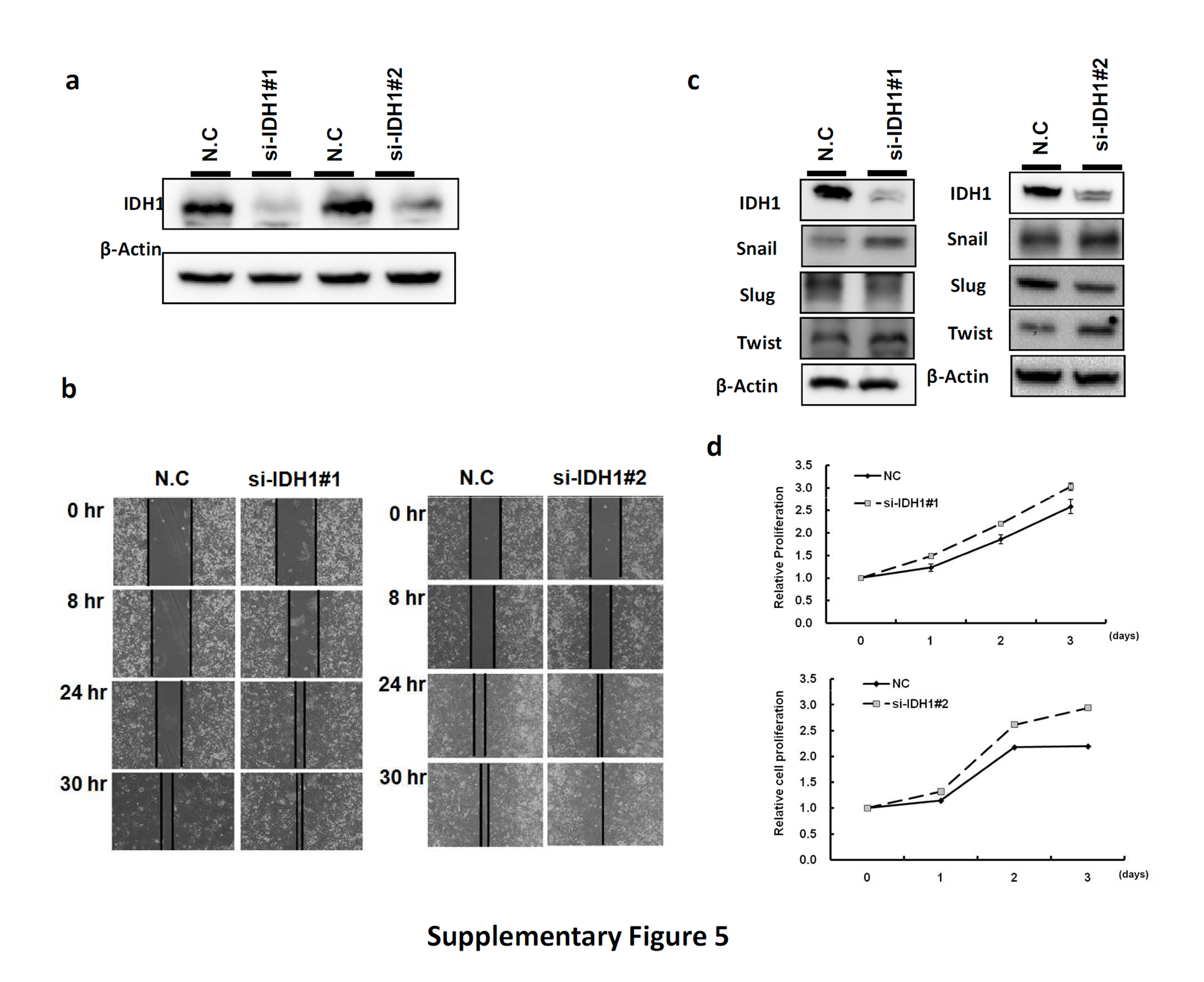

Supplement: Supplementary file 9 — Figure S5. IDH1 knockdown accelerated MCF7 cell proliferation and migration ability. (a) the expression levels of IDH1 were examined in MCF7 cells with siIDH1#1, siIDH1#2, and control transfection through western blotting. (b) A wound healing assay was employed to examine MCF7 cells transfected with siIDH1#1, siIDH1#2, and the scrambled control. (c) The expression levels of IDH1, snail, slug, twist and actin were examined in siIDH1#1, siIDH1#2, and the scrambled control through western blotting. (d) The proliferation assay was performed in MCF-7 cells transfected with the scrambled control, siIDH1#1 and siIDH1#2, respectively. (TIFF 4527 kb) [file 13058_2018_953_MOESM9_ESM.tif]

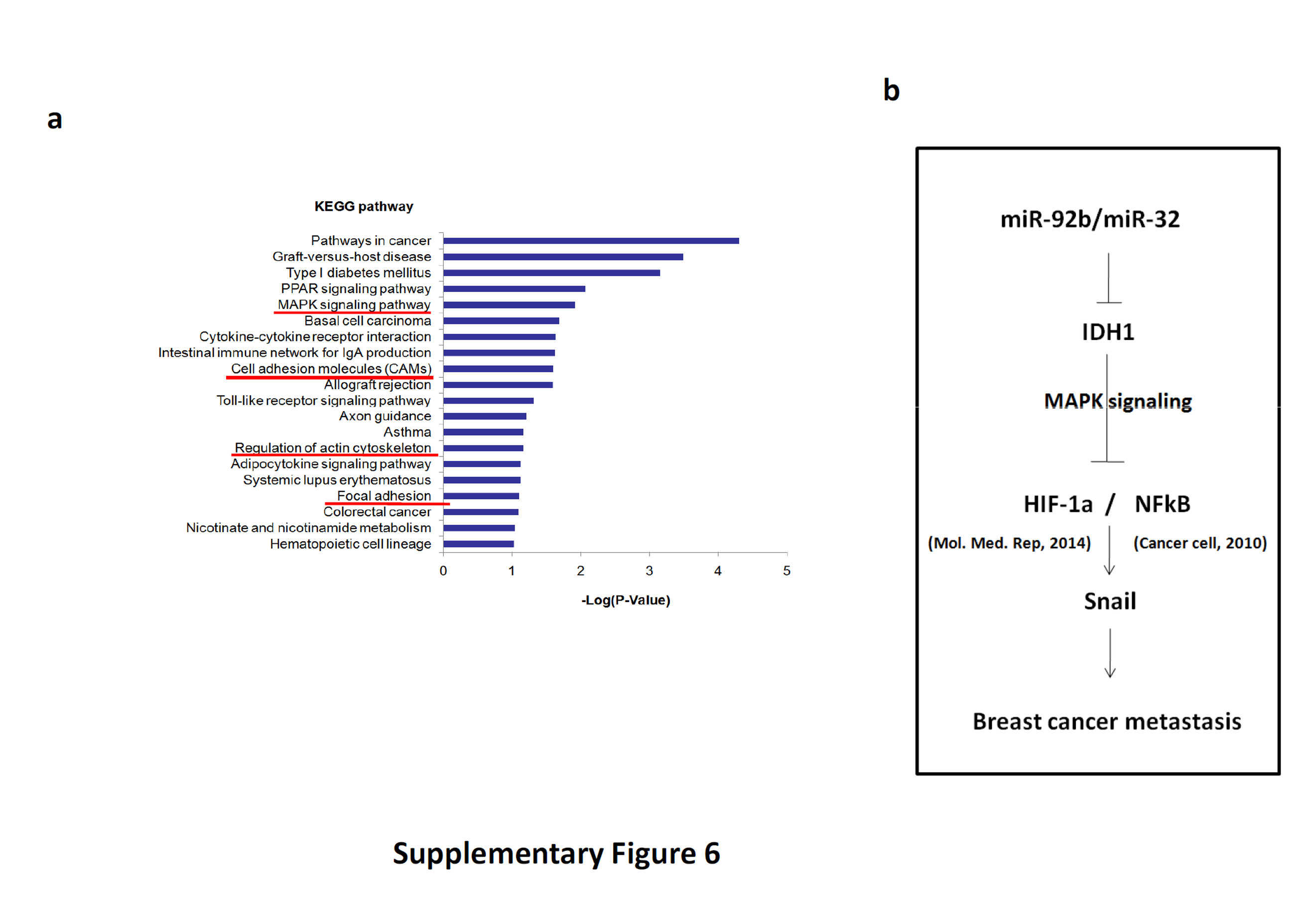

Supplement: Supplementary file 10 — Figure S6. Gene set enrichment analysis for differential expression of genes in MDA-MB-231 cells transfected with si-IDH1 compared with those transfected with scramble control. (a) Differential expression of genes (upregulated or downregulated twofold change) was identified using a microarray approach. These gene sets were significantly enriched in metastasis-associated terms from the Kyoto Encyclopedia of Genes and Genomes. (b) Schematic putative signaling pathway illustrating IDH1-modulated cancer cell invasion. (TIFF 1728 kb) [file 13058_2018_953_MOESM10_ESM.tif]
